# Chemical Probes that Target a Dissociative LuxR-Type Quorum Sensing Receptor in Gram-Negative Bacteria

**DOI:** 10.1021/acschembio.5c00490

**Published:** 2025-09-18

**Authors:** Irene M. Stoutland, Guadalupe Aguirre-Figueroa, Helen E. Blackwell

**Affiliations:** Department of Chemistry, 5228 University of Wisconsin−Madison, 1101 University Avenue, Madison, Wisconsin 53706, United States

## Abstract

Quorum sensing (QS) allows bacteria to respond to changes in cell density and participate in collective behaviors. Interfering with QS could provide a strategy to block pathogenicity, reduce biofouling, and support biotechnology. Many common Gram-negative bacteria use LuxR-type QS receptors that regulate gene transcription in response to *N-*acyl l-homoserine lactone (AHL) signals. The most-studied LuxR-type receptors operate via an associative mechanism, i.e., they dimerize and associate with DNA upon ligand binding. In contrast, members of the less-studied class of dissociative LuxR-type receptors bind DNA as dimers in the absence of a ligand and dissociate from DNA upon ligand binding. Few chemical tools to modulate dissociative receptor activity are known. Such probes could provide new entry into mechanistic studies of LuxI/LuxR-type QS in general. In this report, we describe the discovery of synthetic modulators of EsaR, a dissociative LuxR-type receptor present in the plant pathogen *Pantoea stewartii*, based on AHL scaffolds. Compound activity was evaluated using both cell-based EsaR reporters and a phenotypic assay. We identified compound features associated with agonistic activity in EsaR, some of which were comparable to those of synthetic ligands active in other LuxR-type receptors. However, in contrast to prior studies of AHL mimics, no antagonists were uncovered in EsaR. These results provide chemical strategies to start to investigate mechanisms of ligand response in EsaR and define receptor features driving dissociative vs associative mechanisms in the LuxR-type receptor family. Our findings also suggest that alternate approaches may be required to develop competitive antagonists for dissociative LuxR-type receptors.

## Graphical abstract

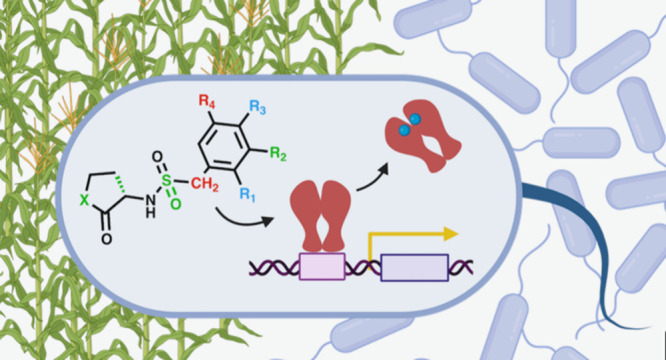

## Introduction

Many common bacteria use quorum sensing (QS) to sense and respond to changes in population density.^1,2^ Each cell produces a basal level of chemical signal, or autoinducer, which increases in concentration with population density. At a threshold concentration, autoinducer binds to a cognate QS receptor, which leads to changes in gene transcription.^3,4^ QS allows bacteria to coordinate group-beneficial phenotypes, such as bioluminescence, biofilm formation, root nodulation, virulence, and secondary metabolite production, at high cell density.^5^ Some bacteria utilize multiple orthogonal QS systems that differ in the nature of the chemical signal and the mechanisms by which it is produced and sensed.^1,4^ The LuxI/LuxR QS system is common in Gram-negative proteobacteria and typically consists of a LuxI-type synthase that produces an *N*-acyl l-homoserine lactone (AHL) autoinducer and a cytoplasmic LuxR-type receptor that regulates transcription based on AHL concentration (Figure 1A).^3^

**Figure 1:**
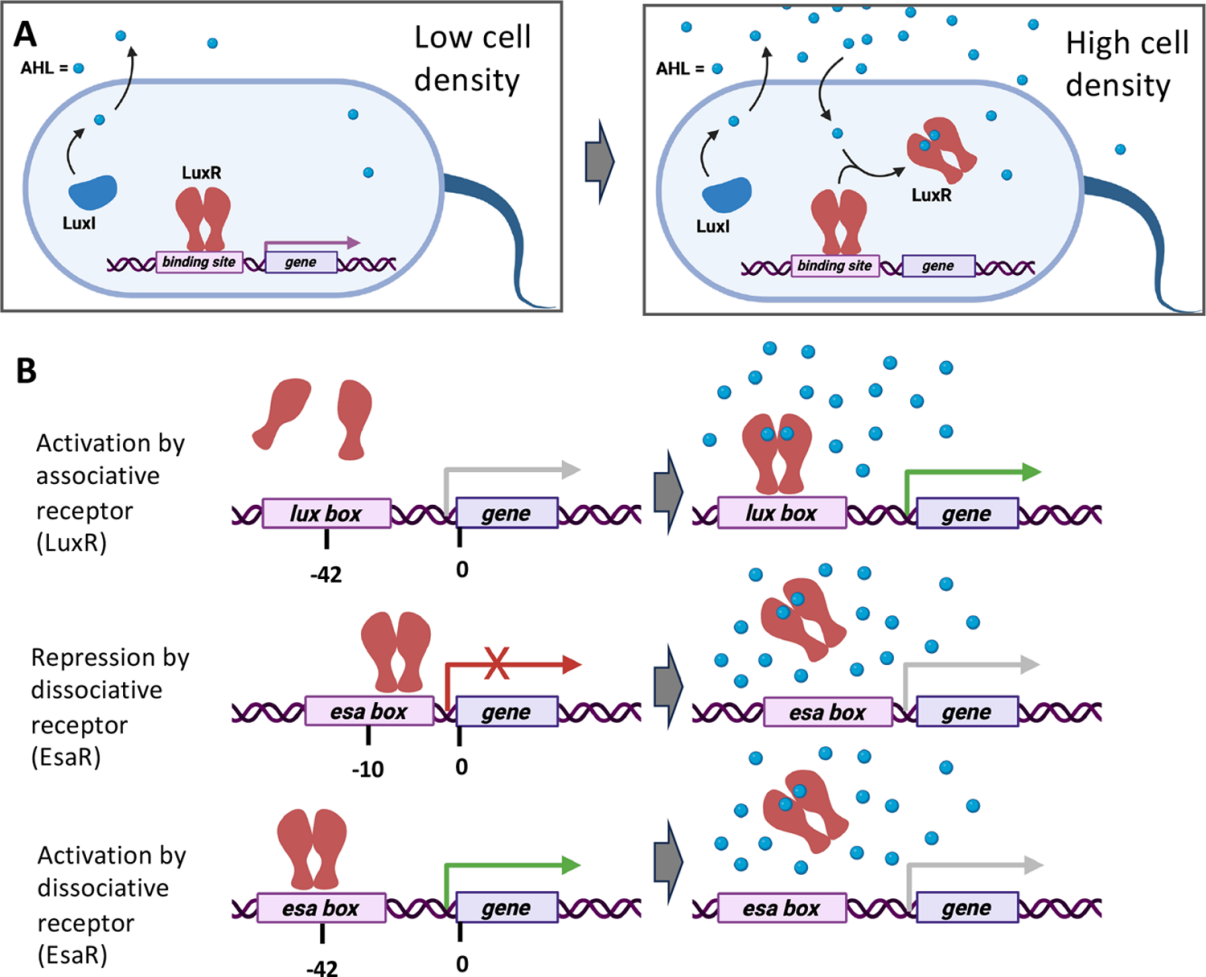
LuxI/LuxR QS pathways and gene regulation. (A) Schematic of LuxI/LuxR QS regulated by a dissociative LuxR-type receptor. A LuxI-type synthase produces an AHL signal at basal levels, and the local AHL concentration increases with cell density. At high AHL concentration, the AHL binds to a LuxR-type receptor, leading to changes in the expression of QS-controlled genes. (B) Associative LuxR-type receptors dimerize and bind to DNA in the presence of AHL (*top*) to activate transcription. Dissociative LuxR-type receptors bind to DNA as dimers to either repress (*middle*) or activate (*bottom*) transcription and dissociate from DNA upon AHL binding. Gray arrows indicate constitutive transcriptional activity, green arrows indicate activation, and red arrow indicates repression.

Of the many LuxR homologues identified to date, the relatively few that have been characterized share some common features but vary considerably in amino acid sequence (∼25% identity^6^) and mode of action. Most well-studied LuxR-type receptors are referred to as “associative” because they remain free monomers in the absence of autoinducer, and dimerize and bind to DNA in the presence of ligand (Figure 1B).^7^ Some associative LuxR homologues, such as TraR from *Agrobacterium tumefaciens* , bind AHL cotranslationally and are rapidly degraded in the absence of ligand.^6^ Others, such as LasR in *Pseudomonas aeruginosa* , bind AHL reversibly but are relatively unstable in its absence.^8^ Still others, such as SdiA in *Escherichia coli* , are relatively stable and correctly folded in the absence of AHL but require ligand to dimerize and bind to DNA.^9^ In contrast to associative LuxR-type receptors, “dissociative” LuxRs bind DNA as dimers in the absence of ligand and dissociate upon autoinducer binding (Figure 1A,B). In vitro biochemical studies of associative LuxRs often have been hampered by the instability and insolubility of these receptors, especially in the absence of ligand.^10^ Because dissociative LuxRs do not require ligand for dimerization and DNA binding, this receptor class may prove more amenable for in vitro biochemical and structural studies in the presence and absence of ligand.

Many questions remain about the biochemical mechanisms of LuxR-type protein activation and inactivation, along with the broader role of QS in bacterial communities. Chemical modulators of QS are valuable tools to interrogate this cell–cell communication mechanism and simultaneously can support applications in antivirulence, antibiofouling, and synthetic biology/bioengineering.^11,12^ Various research groups have targeted QS through inhibition of QS signal synthesis, degradation of QS signals in the environment, or prevention of signal detection by bacteria, with significant contributions reported by Bassler,^12,13^ Greenberg,^14^ Meijler,^15^ Spring,^16^ and others.^17–19^ Our research laboratory has designed and synthesized large collections of non-native AHLs that can strongly modulate LuxR-type receptors (as agonists, partial agonists, or competitive antagonists) from an array of Gram-negative species.^20–24^ These chemical probes have proven useful for the study of bacterial group behaviors ranging from swarming^25^ to toxin production^26^ to symbioses.^27^

Several important plant, animal, and human pathogens harbor dissociative LuxRs, and many of these receptors play a role in regulating virulence,^28^ yet information about the effects of non-native ligands on dissociative LuxR-type receptors is markedly lacking relative to associative LuxRs. Several studies have identified compounds that affect QS-related phenotypes in bacteria that contain dissociative LuxR-type receptors,^29–39^ but few provide experimental evidence of direct interactions of these compounds with a dissociative LuxR receptor.^40^ Compounds may affect QS-regulated phenotypes by altering LuxR or LuxI expression,^41–43^ rather than by direct interaction with the LuxR-type receptor, and the presence of multiple LuxR-type receptors can complicate the interpretation of phenotypic assays for compound activity. To date, the most extensive study of non-native ligands for dissociative LuxR receptors was reported by our laboratory in 2011, in which we identified several modulators of ExpR1 and ExpR2 from the plant pathogen *Pectobacterium versatile* Ecc71 (previously *P. carotovora* subsp. carotovora).^44^ This report revealed AHL analogs with either agonistic or antagonistic activity in both receptors, indicating it was possible to modulate dissociative LuxR-type receptors with synthetic ligands.

In the current study, we sought an alternate dissociative LuxR-type receptor for the development of chemical probes and selected EsaR for investigations because of its well-characterized role in infection and potential to facilitate future biochemical studies. EsaR regulates various phenotypes associated with virulence in the plant pathogen *Pantoea stewartii* subsp. stewartii (*P. stewartii* hereafter) through the direct regulation of at least 16 promoters^45^ and responds most strongly to 3-oxo-hexanoyl HL (OHHL).^46^ *P. stewartii* causes Stewart’s wilt in corn and can cause substantial crop losses in susceptible varieties.^47^ Ligand- and DNA-binding has been well characterized in EsaR, and the receptor is reported to dimerize both in the presence and absence of OHHL.^48^ These features bode well for mechanistic studies to understand ligand:receptor interactions with this receptor, along with exploring EsaR as a potential antivirulence target. A 2020 study by Neupane et al. described several flavonoid modulators of EsaR identified via computational modeling;^49^ their bioactivity, however, is yet to be verified experimentally, and to our knowledge, no other non-native EsaR ligands have been reported.

Herein, we report the evaluation of a library of synthetic AHL analogues for agonism and antagonism in EsaR and the identification of the first synthetic agonists of EsaR. Compound activity was evaluated using transcriptional reporter assays in both *E. coli* and *P. stewartii* and in a phenotypic assay in *P. stewartii*. To start, we investigated the activity of a set of compounds previously identified as agonists and antagonists of other LuxR-type receptors that also respond to OHHL as their native signal (i.e., LuxR in *Vibrio fischeri* and ExpR1 and ExpR2 in *P. versatile* Ecc71).^44^ Our results showed similar trends in agonism in all four receptors, but we observed notable differences in antagonism. Surprisingly, none of the compounds reported to antagonize LuxR, ExpR1, or ExpR2 inhibited EsaR. We expanded our screen and discovered several potent EsaR agonists among a set of sulfonyl HL derivatives. The compounds identified here provide a chemical toolbox for tuning EsaR function and studying its role in *P. stewartii* pathogenicity. These molecules and their activity profiles also point toward paths to start delineating the mechanistic differences between associative and dissociative LuxR-type receptors.

## Results and Discussion

### Compound Selection and Evaluation Strategies

Approximately ∼100 native AHLs and AHL analogs were chosen from our in-house libraries for testing and are shown in Figure 2. EsaR transcriptional activation and repression were examined using *E. coli* and *P. stewartii* reporters (strains and plasmids listed in Table S1; see Methods section for assay details). OHHL binding blocks EsaR’s ability to either activate or repress transcription (Figure 1B); whether EsaR activates or represses a promoter depends on the location of the EsaR binding site.^28^ We reasoned that non-native EsaR agonists would act similarly to OHHL, while EsaR antagonists would be able to compete with but negate the effect of OHHL. We constructed reporters in *E. coli* based on previously reported EsaR reporters (Table S1). Each strain contained an EsaR expression plasmid (pJN105-esaR) and a reporter plasmid with either an EsaR-activated promoter (*pesaS*)^50^ or an EsaR-repressed promoter (*pesaR*)^50^ upstream of luxABCDE or GFP. *E. coli* luciferase reporters were used for initial screening, but certain compounds were found to inhibit luminescence independent of EsaR (Figure S1A), so *E. coli* GFP reporters were used to determine EC_50_ values for these analogues. Although the EsaR repression reporter containing *pesaR* fused to GFP showed relatively high error due to a low dynamic range (Figure S1C), agonism trends were similar at the EsaR-activated and -repressed promoters. The *P. stewartii* transcriptional reporters were constructed in a *P. stewartii* (Δ*esaI)* strain (ESN51) and contained the same GFP reporter plasmids as the *E. coli* reporters (Table S1); these strains could not biosynthesize OHHL but produced EsaR naturally. Using these reporter systems, all compounds were tested for agonism (in the absence of native ligand, OHHL) and competitive antagonism (in the presence of OHHL). Compound activities are listed in Tables 1 and 2. As part of this analysis, we also examined the compounds previously shown computationally by Neupane et al. to bind EsaR^49^ and did not observe any appreciable agonism or antagonism at EsaR (see SI).

**Figure 2:**
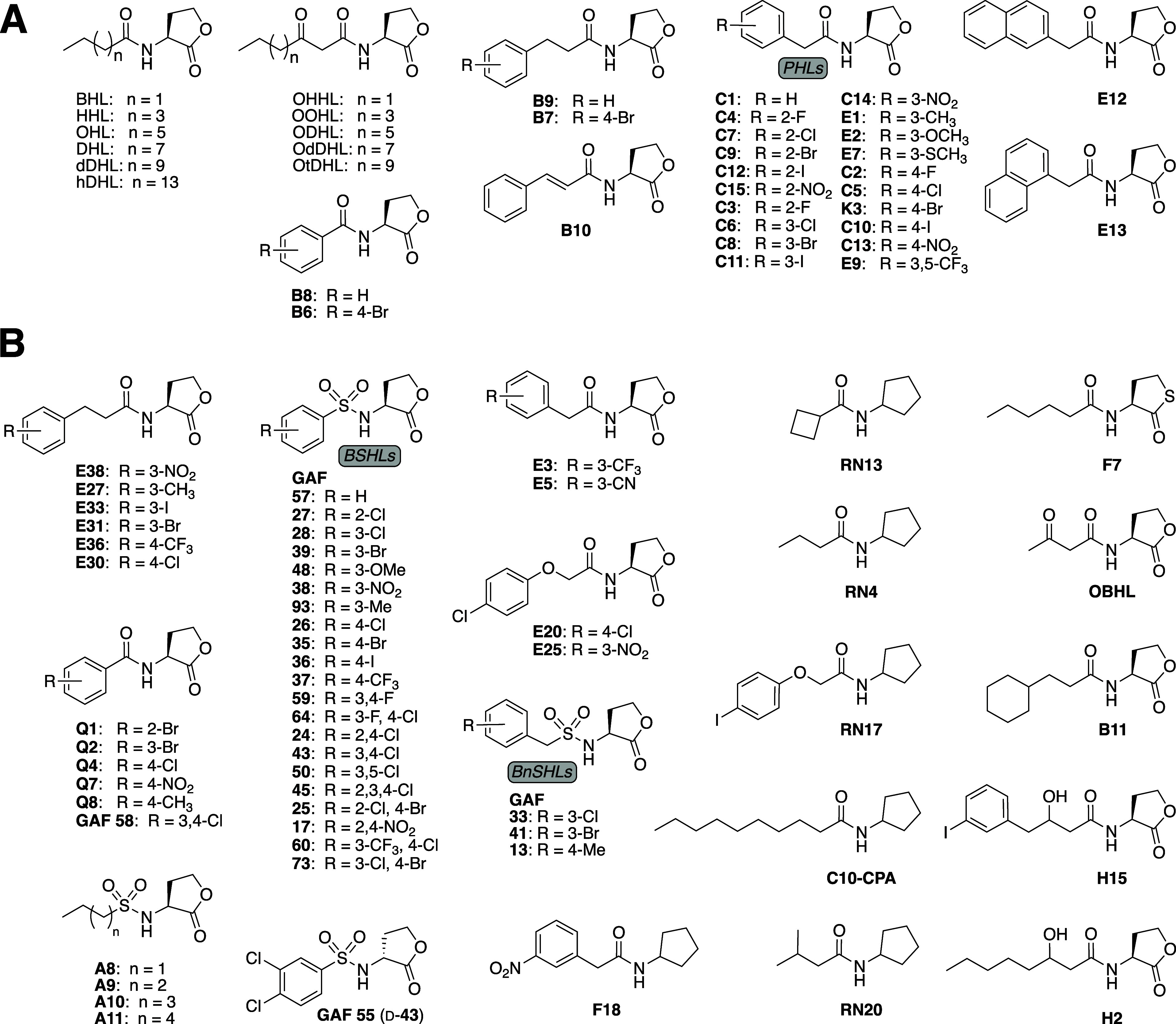
Compounds tested in this study. Native AHLs named via standard abbreviations. Other compounds are numbered according to prior reports from our lab for consistency.^21,51–53^ “GAF” compound numbers match our report on sulfonyl HLs.^54^ (A) Compounds previously screened in LuxR and ExpR1/2.^44^ (B) Second-generation set of compounds screened in EsaR. **H2** and **H15** are diastereomeric mixtures.

**Table 1:**
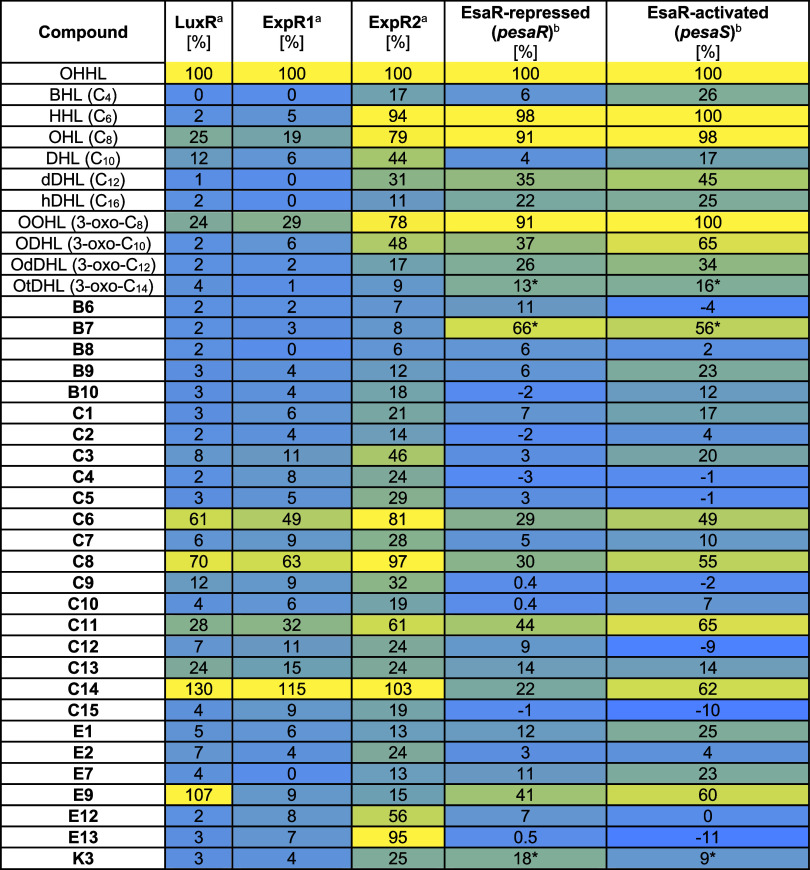
Comparative Agonism Activity Data for the AHLs in Figure 2A in LuxR, ExpR1/2, and EsaR in Cell-Based Reporters^c^

a Data for LuxR and ExpR1/2 are reproduced from ref 44. In that study, compounds were tested at 200 μM. LuxR activation was measured by luminescence in *V. fischeri* ES114 (Δ*luxI*), and ExpR1 and ExpR2 activity was measured by cellulase activity in *P. versatile* (previously *P. carotovora*) AC5117 (Δ*expI:*Δ*expR2*) and *P. versatile* AC5099 (Δ*expI:*Δ*expR1*), respectively.

b Compounds were tested at 100 μM in EsaR. EsaR activation was measured by luciferase activity or GFP production in an *E. coli* reporter; asterisks indicate that GFP reporter was used. For the EsaR-repressed reporter, data are corrected for OD_600_ and normalized to 100 μM OHHL (100%) and DMSO (0%) by taking (raw signal/OD_600_-DMSO)/OHHL. For the EsaR-activated promoter, data are reported as 100 – [(raw signal/OD_600_ – OHHL)/DMSO]. Values represent at least 1 biological and 3 technical replicates. See Methods section for additional assay details.

c Color intensity indicates degree of activation, from high in yellow to low in blue.

**Table 2:**
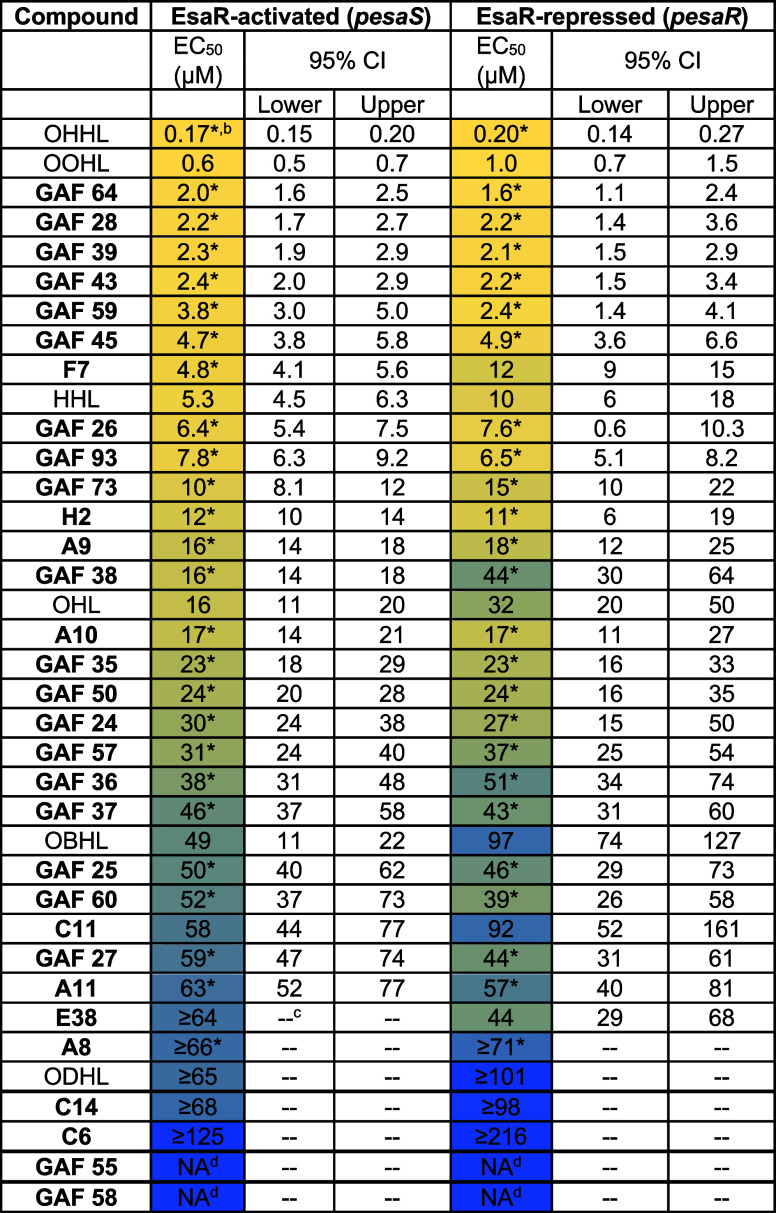
EC_50_ Values for Selected EsaR Agonists in *E. coli* Reporters^a^,^e^

a Data was corrected for OD_600_ and normalized, for the activated promoter, to OHHL (0%) and DMSO (100%) or, for the repressed promoter, to OHHL (100%) and DMSO (0%). CI, confidence interval.

b Asterisk indicates that *E. coli* GFP reporter was used; other values determined in *E. coli* luciferase reporter. Values represent at least 3 biological replicates of 3 technical replicates. See Methods section for details of assays and SI for full dose–response curves.

c “--”, not calculated.

d NA indicates that the compound was inactive or only active at the highest concentration tested (1 mM), and therefore an EC_50_ could not be estimated.

e Color intensity indicates potency, from high in yellow to weak in blue.

### Comparison of Compound Activity in Receptors with Degenerate Native Ligands

We first examined the activity of a set of AHLs reported previously to modulate ExpR1, ExpR2, and LuxR (shown in Figure 2A)^44^ in the EsaR reporter systems and compared activity profiles across the four receptors (Table 1). While LuxR functions via an associative mechanism, ExpR1 and ExpR2 (ExpR1/2 for brevity) are dissociative receptors, and all three respond to OHHL as their native ligand. As EsaR shares OHHL as its native signal, we expected EsaR to show a response similar to that of LuxR and ExpR1/2 to these non-native AHLs. This hypothesis held true for certain compounds: like ExpR2, EsaR was strongly activated by ligands structurally similar to OHHL, namely the native signals hexanoyl HL, octanoyl HL, and 3-(oxo)-octanoyl HL (% activation at 100 μM listed in Table 1). Non-native phenylacetanoyl HLs (PHLs) with electron-withdrawing groups at the *meta* position (**C6**, **C8**, **C11**, and **C14**) were among the strongest non-native agonists in all four receptors. However, while 3-NO_2_ PHL (**C14**) was previously reported to be a superactivator (inducing activation over 100%)^44^ in LuxR and ExpR1 and more potent than OHHL in LuxR, ExpR1, and ExpR2, it acted only as a moderate activator in EsaR. Although several compounds showed potency comparable to OHHL in LuxR and ExpR1/2,^44^ the most potent EsaR agonist of this initial group (3-iodo PHL, **C11**) was ∼300-fold less potent than OHHL (EC_50_ value = 58 vs 0.14 μM, respectively; Table 2). More surprisingly, however, none of the compounds reported to antagonize LuxR, ExpR1, or ExpR2 in these prior studies antagonized EsaR (Figures S2 and S3, Table S3); these compounds were either nearly inactive in EsaR (4-Cl PHL **C5** and 3-SCH_3_ PHL **E7**) or agonized EsaR (octanoyl HL, 3-(oxo)-dodecanoyl HL, 4-Br phenylpropanoyl HL **B7**, and 3-I PHL **C11**) instead. These divergent ligand activities are not wholly unprecedented for this set of receptors, however, given that the LuxR antagonists **E12** and **E13** were also found to act as agonists in ExpR2.^44^

One difference between our prior study in ExpR1/2 and that reported here in EsaR is that the former relied on QS-controlled phenotypes in *P. versatile* Ecc71 (i.e., cellulase and pectate lyase activity)^44^ to measure compound activity, unlike the heterologous transcriptional reporters used for EsaR. To further investigate the differences in antagonism assay data observed between EsaR and ExpR1/2, an *E. coli* reporter was created with an ExpR2 protein production plasmid (pJN105-expR2) and a reporter plasmid with an ExpR2-activated promoter (*prsmA*) controlling GFP (see Methods section).^55^ As expected, OHHL and 3-NO_2_ PHL **C14** acted as agonists (Figure S4A,C), although PHL **C14** was approximately 40-fold less potent than OHHL, in contrast to our previous report suggesting that **C14** was a more potent ExpR2 agonist than OHHL.^44^ Several compounds previously shown to inhibit ExpR2-dependent phenotypes were tested in the *E. coli* reporter and failed to show antagonism (Figure S4B,D). Furthermore, 4-Br phenylpropanoyl HL **B7** now behaved as an agonist in the ExpR2 *E. coli* reporter, analogous to its profile in the EsaR *E. coli* reporter. We return to possible explanations for the differences observed between the ExpR2 phenotypic assays and transcriptional reporters below.

### Identification of Potent EsaR Agonists

Because few of the non-native compounds previously tested in ExpR1/2 and LuxR showed strong activity in EsaR, a second round of compounds from our in-house AHL analog library was selected for screening (Figure 2B). Many were chosen based on structural similarity to compounds that displayed agonistic activity, albeit modest, in EsaR in the first round. Representative results of the primary, single concentration screen (at 100 μM) are shown in Figures 3 and S5 and revealed several potent EsaR agonists, most notably a group of AHL analogs containing a sulfonamide linkage between the tail group and the HL headgroup, as opposed to the amide linkage in native AHLs.^54^ No EsaR antagonists were identified again, however.

**Figure 3:**
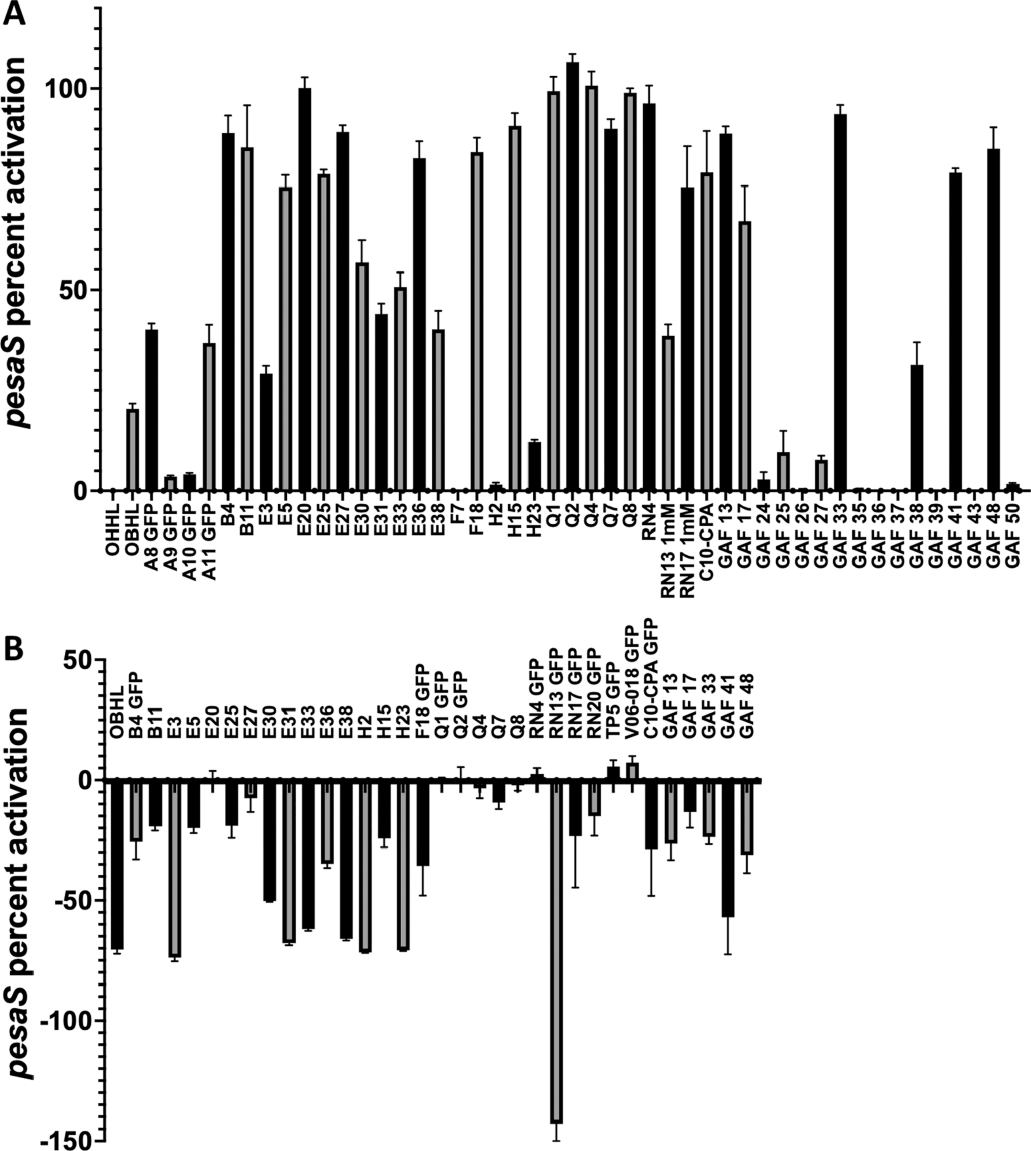
Representative agonism and antagonism primary screening data for the second round of compounds (Figure 2B) in the EsaR-activated reporter. Compound names listed on the *x*-axes; all compounds tested at 100 μM (unless indicated next to compound name). Data obtained in the *E. coli* luciferase reporter (unless GFP is indicated next to compound name); all data corrected for OD_600_. Bars alternate in black and gray for clarity only. See Methods section for experimental details. (A) Agonism data. Data normalized to 100 μM OHHL (0%) and DMSO (100%). (B) Antagonism data. Selected compounds were competed against 180 nM OHHL (approximate EC_50_). Data normalized to 180 nM OHHL (0%) and DMSO (100%). Negative values indicate activators. Values represent at least 1 biological and 3 technical replicates. Error bars indicate standard deviation. The known LasR antagonists TP-5 ^14^ and V-06–018 ^56^ were included for comparison and found to be inactive as EsaR antagonists. See Figure S5 for similar screening data in the EsaR-repressed reporter.

For compounds showing greater than 50% agonistic activity in the first or second round primary screens, EC_50_ values were determined using the *E. coli* EsaR reporters (listed in Table 2). All compounds with dose–response curves that leveled off within the concentration range tested reached 100% EsaR activation (see SI for dose–response curves). This result is surprising, as we have frequently identified many compounds with maximum efficacies lower than that of the native ligand (i.e., partial agonists) in similar studies of other LuxR-type receptors.^57^ Certain sulfonyl HLs were found to inhibit luminescence at high concentration (Figure S1A), so these compounds were examined in GFP reporters (see above). Luciferase and GFP reporters for EsaR in *E. coli* produced similar dose–response curves with OHHL (Figure S1B). For analysis of EC_50_ values, data from the EsaR-activated reporter were used, as this reporter had high dynamic range and, as a result, relatively low variation.

Overall, the most potent EsaR agonists were sulfonyl HLs with aryl tails (Figure 2), with EC_50_ values in the single-digit micromolar range (2.0–4.7 μM; Table 2). A summary of the structure–activity relationships (SARs) for EsaR agonism by aryl sulfonyl HLs is provided in Figure 4. Interestingly, while the most active AHL analogs in the initial screen were PHLs (**C6**, **C8**, **C11**, **C14**, and **E9**; Table 1), the homologous benzyl sulfonyl HLs (BnSHLs **13**, **33**, and **41**; Figure 2B) could not agonize EsaR more than 50% at 100 μM (Figure 3). The one-carbon-shorter homologues—i.e., benzenesulfonyl HLs (BSHLs) with *meta* halogen substituents—were the strongest EsaR agonists identified in this study (**GAF 28**, **39**, **43**, **64**, and **59**; Table 2). A *para*-Cl was more agonizing than no substituents at all (**GAF 26** vs **57**), but in general, *ortho* and *para* substituents either had minimal effects on potency (i.e., **GAF 24**, **25**, **27**, **35**, **36, 43**, **64**, and **59**) or decreased potency (**GAF 45**, **50**, and **73**). Given the similar atomic radii of Cl and methyl substituents, the intermediate activity of *meta* methyl BSHL **GAF 93** suggests substituent size is one of the factors determining compound potency. A *meta* NO_2_ slightly increased the potency of the BSHL scaffold (**GAF 38**), as also observed with the *meta* NO_2_ PHL (**C14**; Table 1).

**Figure 4:**
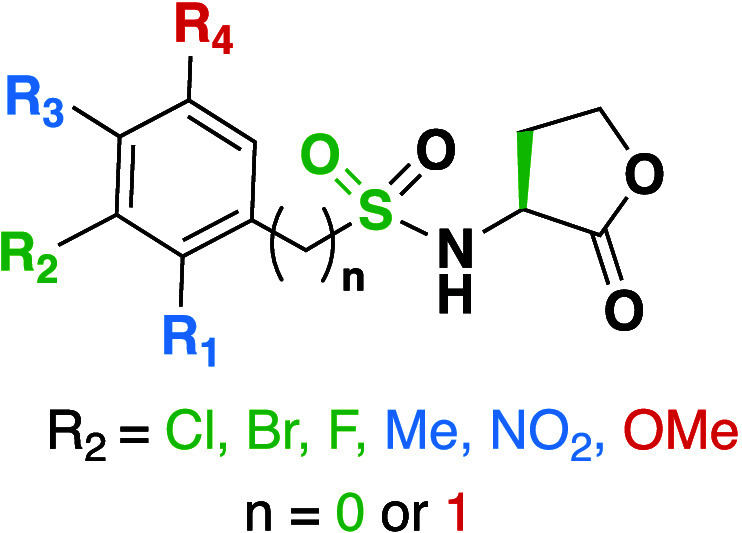
Summary of key SAR trends for EsaR agonists identified herein. Green moieties enhanced potency (decreased EC_50_), red moieties decrease potency (increased EC_50_), and blue moieties had minimal effect on potency. The green S = O indicates that the sulfonamide linker yields a more potent compound than an amide linker.

Changes to other BSHL functionalities had large and negative effects on compound activity in EsaR. Modifying one of the most potent BSHL modulators (3,4-dichloro BSHL, **GAF 43**) by replacing the sulfonyl with a carbonyl or inverting the stereochemistry of the HL produced compounds that were less than 50% active at 100 μM (**GAF 58** and **55**; Table 2). The substituted phenyl ring was also important for activity: alkyl sulfonyl HLs with 4- or 5- carbon tails were more activating than a BSHL with an unsubstituted phenyl ring (**A9** and **A10** vs **GAF 57**; Table 2), but less activating than the top EsaR agonists (e.g., 3-F, 4-Cl BSHL (**GAF 64**), EC_50_ = 2.0 μM). Note that sulfonyl HL **A10** resembles hexanoyl HL, as they share a 6-atom tail, yet **A10** is about 3-fold less potent than hexanoyl HL (Table 2). These results indicate that the combination of the sulfonyl moiety, l-HL headgroup, and phenyl tail contributes to the potency of the top sulfonyl HL agonists in EsaR (Figure 4).

Among the additional AHLs screened for EsaR activity, **H2**, which resembles OOHL with the 3-oxo group replaced with a 3-hydroxy, was ∼20-fold less potent than OOHL (Table 2), suggestive that the oxidation state at this position is important for EsaR agonism. In addition, several cyclopentylamide compounds (**C10-CPA**, **RN4**, **RN17**, **F18**, **RN20**, and **RN13**; Figure 2B) were tested for EsaR antagonism, given that AHL analogs with this headgroup have been shown to inhibit other LuxR-type receptors.^58^ None of these compounds antagonized EsaR. Instead, **RN13** was a moderate agonist of EsaR (Figure 3), albeit at high concentration, which suggests that the HL headgroup may not be essential for EsaR activation.

### Evaluation of Compounds for Activity in *P. stewartii*

We were curious whether the EsaR agonists identified in the *E. coli* reporters were also active in EsaR’s native host organism. In past studies, some *E. coli* LuxR-type receptor reporters have yielded EC_50_ values for exogenous ligands that differ by orders of magnitude relative to reporters constructed in native bacteria.^59^ Overexpression of LuxR-type receptors in *E. coli* has been proposed to lead to increased DNA binding and an overestimation of associative receptor sensitivity to agonists, as measured with lower EC_50_ values.^24^ However, because dissociative LuxR-type receptors bind to DNA in the absence of ligand, high receptor expression could instead lead to an underestimate of agonism. To account for any unintended effects of the *E. coli* genetic background and test compound activity at native levels of EsaR, we created genetic reporters in *P. stewartii* (see above and Methods section for details). In the *P. stewartii* reporters, the EC_50_ values for OHHL, the strong agonist BSHL **GAF 64**, and the less potent agonist BSHL **GAF 35** were largely comparable to the corresponding *E. coli* reporters (Table 3). These data suggest that the *E. coli* reporters provide a reasonably accurate gauge of compound efficacy and potency in EsaR relative to the *P. stewartii* reporter. Slight growth inhibition was observed for some compounds in the *P. stewartii* reporters but not in *E. coli* , and the compounds were active in EsaR well below concentrations that affect cell growth in *P. stewartii* (Figure S1D). Since the previously reported ExpR1/2 antagonists did not show any EsaR antagonism in the *E. coli* reporters (see above), we tested these compounds in the *P. stewartii* reporters to verify that the observed lack of antagonism was not an artifact of the reporter system. No antagonism was observed in *P. stewartii* (Figure S3), again indicating that the *E. coli* and *P. stewartii* reporter activity data aligned for this set of compounds.

**Table 3:** Comparative Agonism Activity Data for Selected Compounds in *E. coli* and *P. stewartii* EsaR Reporters^a^

	***E. coli***						***P. stewartii*** **ESN51**					
	*pesaS*			*pesaR*			*pesaS*			*pesaR*		
	**EC**_**50**_ **(μM)**	95% CI		**EC**_**50**_ **(μM)**	95% CI		**EC**_**50**_ **(μM)**	95% CI		**EC**_**50**_ **(μM)**	95% CI	
compound		lower	upper		lower	upper		lower	upper		lower	upper
OHHL	**0.17**	0.15	0.20	**0.20**	0.14	0.27	**0.12**	0.09	0.14	**0.16**	0.12	0.21
**GAF 64**	**2.0**	1.6	2.5	**1.6**	1.1	2.4	**2.7**	2.3	3.3	**6.7**	5.3	8.5
**GAF 35**	**23**	18	29	**23**	16	33	**24**	18	31	**27**	21	34

a Data was corrected for OD_600_ and normalized, for the activated promoter, to OHHL (0%) and DMSO (100%) or, for the repressed promoter, to OHHL (100%) and DMSO (0%). Data represents the average of 3 biological replicates, each performed as 3 technical replicates. CI, confidence interval. See Methods section for additional details of assays.

### Phenotypic Assay in *P. stewartii*

In *P. stewartii*, EsaR regulates the production of the exopolysaccharide stewartan, which clogs the xylem of infected plants and leads to the wilt and lesions characteristic of Stewart’s wilt.^60^ EsaR binds directly to the *rcsA* promoter to repress transcription at low cell density, and when this repression is relieved at high cell density, RcsA contributes to activation of the *cps* gene cluster, which encodes the stewartan biosynthetic pathway.^61^ Exopolysaccharide production can be visualized by the growth of mucoid colonies on casamino acid-peptone-glucose (CPG) agar plates, and this phenotypic assay is commonly used to evaluate EsaR-mediated QS in *P. stewartii*.^60,61^ To investigate the ability of the synthetic EsaR agonists uncovered here to regulate a QS-controlled phenotype in *P. stewartii*, selected compounds were tested for their ability to induce mucoidy in the *P. stewartii* (Δ*esaI)* strain, ESN51 (Figure 5). ESN51 mutant colonies appeared rough and separate in the absence of exogenous ligand, but bacterial growth was smooth on agar supplemented with 10 μM OHHL, indicating high exopolysaccharide production. Like the native ligand, the strong EsaR agonist BSHL **GAF 28** induced mucoidy at 10 μM. The less potent agonists **H2** and **GAF 57** induced mucoidy at 100 μM but not at 10 μM, and the very weakly active or inactive compounds **GAF 55** and **A11** did not induce mucoidy. These results are consistent with the agonism trends observed in cell-based reporters for this set of compounds and confirm that these synthetic EsaR agonists can modulate a QS-regulated phenotype in *P. stewartii*.

**Figure 5:**
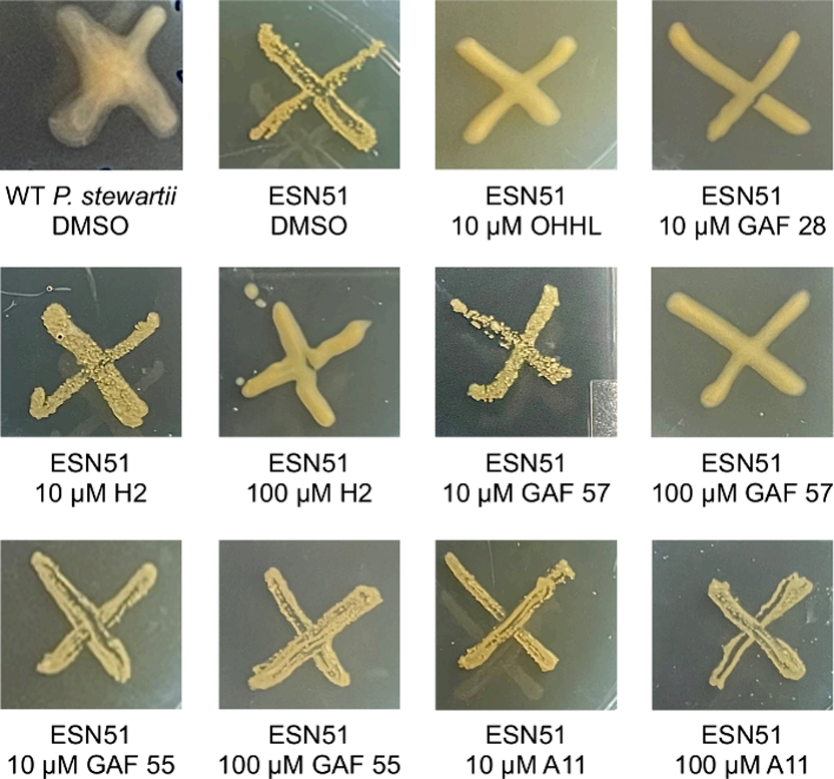
Images of colony mucoidy induced by EsaR agonists in wild type (WT) or Δ*esaI* (ESN51) *P. stewartii* grown on agar. In the absence of agonist, ESN51 mutant colonies appear rough and separate, but in the presence of an agonist, bacterial growth is smooth and individual colonies are invisible. Images are representative of at least 2 biological replicates. See Methods section for assay details.

### Summary and Conclusions

Much remains to be learned about the molecular mechanisms of LuxI/LuxR-type QS and their roles in virulence and in complex, natural environments. Despite extensive work over the past 25+ years on the development of synthetic modulators of associative LuxR-type receptors, little is known about the mechanisms by which dissociative LuxR-type receptors respond to native and synthetic ligands. The current study starts to address this void and identified synthetic agonists with a range of potencies for a dissociative LuxR-type receptor, EsaR. We confirmed the agonistic activities in *E. coli* and *P. stewartii* genetic reporters and in a QS-dependent phenotypic assay in *P. stewartii* linked to virulence. Our data allowed us to delineate the first SARs governing non-native compound activity in EsaR: in general, benzenesulfonyl HLs (BSHLs) with a *meta-*substituted aryl tail were the most active, with EC_50_ values only 10-fold higher than that of EsaR’s native ligand, OHHL.

Our prior study revealed synthetic antagonists of another dissociative receptor, ExpR2, using a phenotypic reporter in *P. versatile* Ecc71,^44^ but in the current study, we found that those compounds no longer acted as ExpR2 antagonists in a heterologous transcriptional reporter. Potential explanations that reconcile these differences include: (1) ExpR2 regulates cellulase and pectate lyase through a different promoter in addition to *prsmA* and responds to the antagonists at this other promoter, or (2) the compounds affect cellulase and pectate lyase activity via a mechanism other than direct regulation by ExpR2. It is also possible that other factors, such as additional protein partners,^62–64^ are involved in ExpR2 regulation and contribute to the observed effects of some compounds. If nonspecific factors were involved, however, it would be surprising to observe the shared trends in antagonism between LuxR and ExpR2. Overall, our results suggest that the lack of antagonism observed in EsaR is not due to mechanistic differences between EsaR and ExpR2, which share 53% sequence identity (see Figure S7 for sequence alignment). While there are not enough studies of modulators of dissociative receptors to determine whether the lack of EsaR and ExpR2 antagonists is unusual among dissociative receptors, we are unaware of reports of associative LuxR-type receptors for which no antagonists have been identified upon targeted screening, even in screens of small sets of compounds.

Future work in the short term will interrogate the abilities of synthetic agonists with different potencies to alter the stability of EsaR in vitro and investigate the origins of stronger agonism by BSHLs relative to structurally similar AHLs. Overall, we anticipate that the EsaR agonists identified here will be useful probes to characterize EsaR function and the chemical features important for productive ligand:receptor interactions, investigate the mechanisms of ligand response and signal transduction from ligand-binding domain to DNA-binding domain (pathways we have begun to study in detail in the associative receptor, QscR),^24^ and elucidate fundamental mechanistic differences between associative and dissociative LuxR-type receptors in general. These and related studies are ongoing and will be reported in due course.

## Methods

### General Reagent and Instrumentation Information

All standard chemical and biological reagents, media, naturally occurring AHLs, and solvents were purchased from commercial sources and used without further purification. Water (18 MΩ) was purified with a Sartorius Arium Pro system. Antibiotics stocks were prepared at 1000× and stored at −20 °C. Luminescence and fluorescence data were collected on a BioTek Synergy 2 plate reader using Gen5 software (version 3.12). Compound stock solutions were prepared in DMSO from samples previously synthesized in our laboratory or by collaborators^51,54,57,65,66^ and stored at −20 °C.

### Bacteriology

The bacteria and plasmids used in this study are listed in Table S1. Strains were stored in 25% glycerol at −80 °C. Overnight cultures were grown in 3–5 mL LB medium with appropriate antibiotics and incubated at 37 °C with shaking (200 rpm) for 16 h.

### Plasmid and Reporter Strain Construction for Compound Testing

Gibson assembly was used to create all constructs. Primers are listed in Table S2. Plasmid pJN105-esaR was created by amplifying *esaR* from pAC-esaR^67^ and inserting it into pJN105. pesaSlux was created by inserting *pesaS*, amplified from pPesaR-AC:gfp,^50^ into the pCS-pesaRlux backbone.^67^ The *pesaR* promoter was amplified from pCS-pesaRlux^67^ and inserted into the pPesaR-AC:gfp backbone^50^ to create pesaR:GFP. *P. stewartii* reporter strains were created by transforming pesaR:GFP or pPesaR-AC:gfp into ESN51 ^60^ by conjugation using *E. coli* S17 λpir as the donor strain. See SI for details of *E. coli* ExpR2 reporter construction.

### *E. coli* Reporter Assay Protocol

Overnight cultures were diluted 1:10 in LB medium with appropriate antibiotics and grown at 37 °C (200 rpm) to an OD_600_ of 0.22–0.27 before induction with 4 mg mL^–1^ arabinose. OD_600_ was measured by placing 200 μL of culture into a 96-well plate and reading optical density using the BioTek Synergy 2 plate reader. Assays were performed in technical triplicate in clear-bottom, white and clear-bottom, black 96-well microtiter plates (Corning Costar 3903 and 3904) for luminescence and fluorescence reporters, respectively. Compounds were dissolved in DMSO, and 2 μL of this stock, or DMSO as a negative control, was added to each well prior to adding 198 μL of induced culture. Plates were incubated at 37 °C (200 rpm) for 3.5 h. Each well was mixed by pipetting before reading OD_600_ and either luminescence or fluorescence. Single-point screens were performed with one or two biological replicates, and all dose–response curves were performed in biological triplicate. EC_50_ values were calculated based on a nonlinear regression of log­(concentration) vs response (three parameters) using GraphPad Prism 9 software.

### *P. stewartii* Reporter Assay Protocol

Compound stocks were prepared and plated as described for *E. coli* reporter assays. Single colonies of ENS51 pPesaR-AC:gfp or ENS51 pesaR:GFP were used to inoculate LB medium supplemented with appropriate antibiotics, and these cultures were grown for 24 h at 28 °C (200 rpm). Cultures were diluted to an OD_600_ of 0.05, and 198 μL was added to each well of a clear-bottom, black 96-well plate and incubated for 6 h at 28 °C (200 rpm) before reading fluorescence and OD_600_.

### Mucoidy Assay Protocol

*P. stewartii* *ΔesaI* (ESN51) was grown on Casamino Peptone Glucose (CPG) agar (1 g/L casamino acids, 10 g/L peptone, 10 g/L glucose, 17 g/L agar) supplemented with a 1:1000 dilution of compound stocks or DMSO. Plates were incubated at 28 °C for 2 days without shaking before imaging with a smartphone camera (Apple iPhone X). Images are representative of at least two biological replicates.

## Supplementary Material



## Notes

Strain and primer information, additional methods, and reporter assay data (PDF)
