# Correction to “Chemical Probes That Target a Dissociative LuxR-Type Quorum Sensing Receptor in Gram-Negative Bacteria”

**DOI:** 10.1021/acschembio.5c00839

**Published:** 2025-11-12

**Authors:** Irene M. Stoutland, Guadalupe Aguirre-Figueroa, Helen E. Blackwell

**Affiliations:** Department of Chemistry, 5228 University of Wisconsin−Madison, 1101 University Ave., Madison, Wisconsin 53706, United States

Figure 2 contains three minor errors. The structure of compound **GAF 64** is incorrect in Figure 2 and as described in the text. The correct structure has 3-Cl, 4-F aryl substituents and is shown in the corrected Figure 2 below. Accordingly, the sentence that describes **GAF 64** on page 2457 of the article should read, “The substituted phenyl ring was also important for activity: alkyl sulfonyl HLs with 4- or 5- carbon tails were more activating than a BSHL with an unsubstituted phenyl ring (**A9** and **A10** vs **GAF 57**; Table 2), but less activating than the top EsaR agonists (e.g., 3-Cl, 4-F BSHL (**GAF 64**), EC_50_ = 2.0 μM).” The structure of compound **C3** is also incorrect in Figure 2 and has a 3-F, rather than a 2-F aryl substituent, as shown in the corrected Figure 2 below. Two citations were erroneously omitted in the Figure 2 legend concerning previously synthesized compounds in Figure 2 (listed below). The first was included in the original article (as ref 66) but not in direct reference to compound origins and should be placed at the end of the text in section (B) of the legend. The second was not included in the original article, describes the synthesis of the E library, and should be placed after the sentence ending in “... consistency” in the legend. None of these errors alter the conclusions of the study.

**Figure 2:**
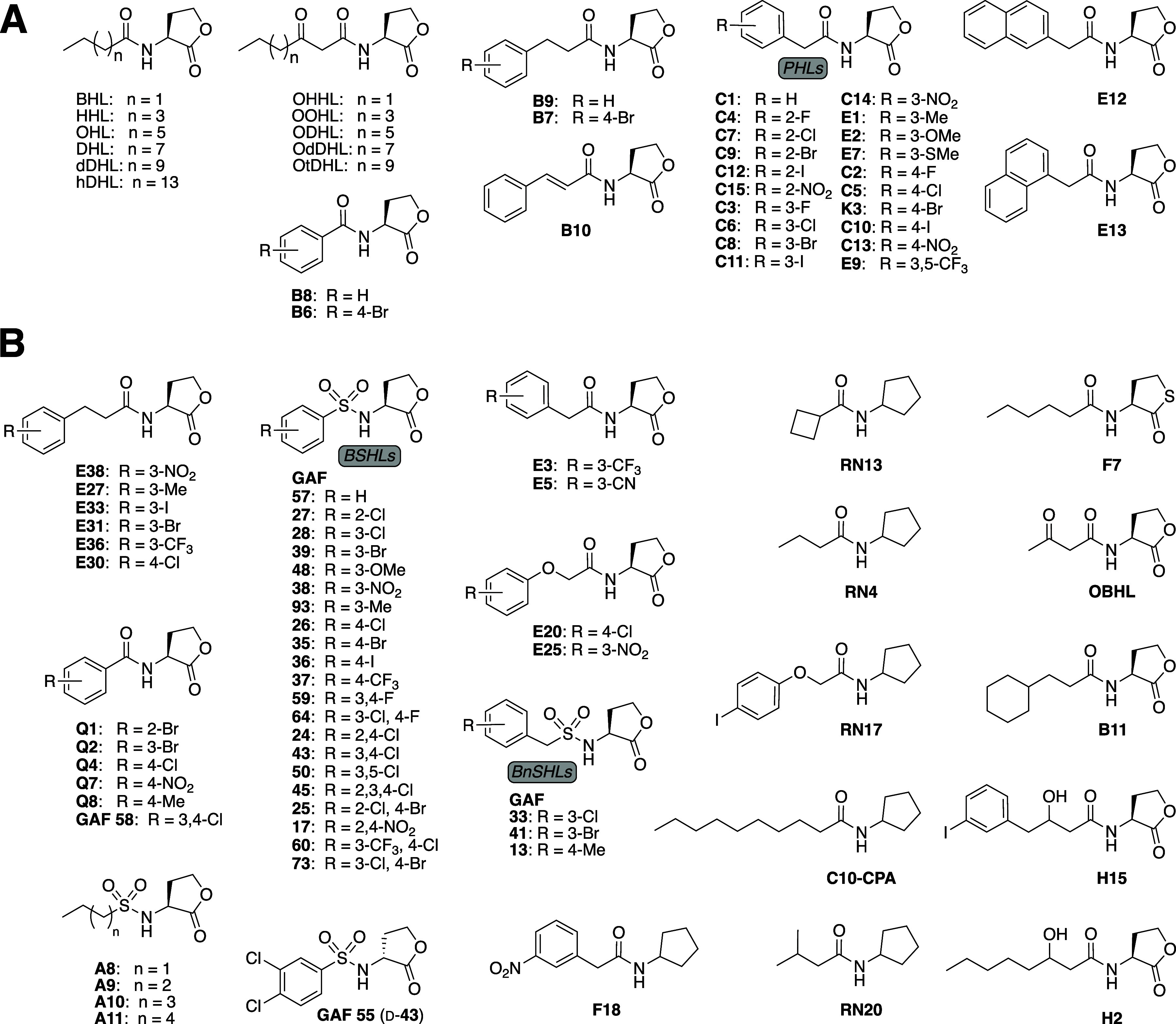
Compounds tested in this study. Native AHLs named via standard abbreviations. Other compounds are numbered according to prior reports from our lab for consistency.^21,51–53^ “GAF” compound numbers match our report on sulfonyl HLs.^54^ (A) Compounds previously screened in LuxR and ExpR1/2.^44^ (B) Second-generation set of compounds screened in EsaR. **H2** and **H15** are diastereomeric mixtures.^1,2^
