# Supplementary material for: Chemical Probes that Target a Dissociative LuxR-Type Quorum Sensing Receptor in Gram-Negative Bacteria
Source: ACS Chem Biol. 2025 Sep 18;20(10):2451–61. doi: 10.1021/acschembio.5c00490 (PMC12459020; doi:10.1021/acschembio.5c00490)
Supplement: Supplementary file 1 [file cb5c00490_si_001.pdf]

SUPPORTING INFORMATION

Chemical probes that target a dissociative LuxR-type quorum sensing receptor in Gram-negative bacteria

Irene M. Stoutland, Guadalupe Aguirre-Figueroa, and Helen E. Blackwell*

*Department of Chemistry, University of Wisconsin–Madison, 1101 University Ave., Madison, WI 53706, USA; *Corresponding author: blackwell@chem.wisc.edu*

CONTENTS.

- Additional experimental methods
- **Table S1.** Bacterial strains and plasmids used in this study
- **Table S2.** Primers used in this study
- **Table S3.** Comparative antagonism assay data for AHLs in four receptors (LuxR, ExpR1, ExpR2, and EsaR)
- **Figure S1.** Luminescence inhibition, dynamic range, and growth of reporter strains
- **Figure S2.** Agonism and antagonism primary screening data in EsaR for compounds previously tested in LuxR, ExpR1, and ExpR2 (data for **Tables 1** and **S3**)
- **Figure S3.** Antagonism dose-response data for selected compounds in *E. coli* GFP and *P. stewartii* EsaR reporters
- **Figure S4.** Screening data in *E. coli* reporter with ExpR2-activated promoter
- **Figure S5.** Agonism and antagonism primary screening data in *E. coli* reporter with EsaR-repressed promoter.
- **Figure S6.** Evaluation of flavonoid compounds predicted by Neupane *et al.* to modulate EsaR in the *P. stewartii pesaS* reporter
- **Figure S7.** Sequence alignment of EsaR and ExpR2
- Dose-response agonism data in *E. coli* EsaR reporters
- References

Additional experimental methods.

Plasmid and reporter strain construction for ExpR2 reporters

Gibson assembly was used to create all constructs. Strains, plasmids and primers are listed in **Tables S1** and **S2**. Plasmid pJN105-expR2 was created by amplifying *expR2* from *Pectobacterium versatile* Ecc71 genomic DNA and inserting it into pJN105, amplified from pJN105-esaR. To create prsmA:GFP, the *rsmA* promoter[1] (nucleotides -99 to +17) was amplified from *P. versatile* Ecc71 genomic DNA and inserted into the pPesaR-AC:gfp backbone. *E. coli* reporter strains were created by transforming pJN105-expR2 and prsmA:GFP into electrocompetent *E. coli* JLD271.

E. coli ExpR2 reporter assay

Single colonies of the reporter strain were used to inoculate LB medium supplemented with appropriate antibiotics, and these cultures were grown overnight at 37 °C with shaking at 200 rpm. Overnight cultures were diluted 1:10 in LB medium with appropriate antibiotics and grown at 37 °C to an OD_{600} of 0.22–0.27 before induction with 0.4 mg/mL arabinose. Arabinose concentration was optimized to maximize dynamic range of reporter data. Compounds were dissolved in DMSO, and 2 µL of this stock, or DMSO as a negative control, was added to each well of a clear-bottom, black 96-well plate prior to adding 198 µL of induced culture. Plates were incubated at 37 °C with shaking at 200 rpm for 3.5 hr. Assays were performed in technical and biological triplicate. Each well was mixed by pipetting before reading fluorescence and OD_{600}. EC_{50} values were calculated based on a nonlinear regression of log(concentration) vs. response - variable slope (four parameters) using GraphPad Prism 9 software.

Table S1. Bacterial strains and plasmids use in this study.

**Plasmids**	**Description**	**Source**
pAC-esaR	*esaR* under control of *pLac* promoter (Cm^R)	[2], Addgene
pPesaR-AC:gfp	WT p*esaS* promoter from *Pantoea stewartii* subsp. *stewartii* (*P. stewartii* hereafter) controlling *GFP* (Ap^R)	[3]
pesaR:GFP	WT p*esaR* promoter from *P. stewartii* controlling *GFP* (Ap^R)	This study
pCS-pesaRlux	WT p*esaR* promoter from *P. stewartii* controlling *luxCDABE* (Km^R)	[2], Addgene
pesaSlux	WT p*esaS* promoter from *P. stewartii* controlling *luxCDABE* (Km^R)	This study (based on [2])
pJN105-esaR	pJN105 with arabinose-inducible promoter controlling *esaR* (Gm^R)	This study
pJN105-expR2	pJN105 with arabinose-inducible promoter controlling *expR2* (Gm^R)	This study
prsmA:GFP	WT *rsmA* promoter from *Pectobacterium versatile* Ecc71 controlling *GFP* (Ap^R)	This study
**Strains**		
JLD271	*E. coli* K12 Δ*sdiA*ec (Cm^R)	[4]
S17 λpir	*E. coli TpR SmR recA, thi, pro, hsdR-M+RP4: 2-Tc:Mu: Km Tn7 λpir*	[5]
DC283	WT *P. stewartii* SS104 (Nal^R)	[3]
ESN51	DC283 *ΔesaI* mutant (*esaI*::Tn5seqN51)	[3]
JLD271 pJN105-esaR, pCS-pesaRlux	*E. coli* luciferase reporter for EsaR transcriptional repression	This study
JLD271 pJN105-esaR, pesaSlux	*E. coli* luciferase reporter for EsaR transcriptional activation	This study
JLD271 pJN105-esaR, pesaR-GFP	*E. coli* fluorescence reporter for EsaR transcriptional repression	This study
JLD271 pJN105-esaR, pPesaR-AC:gfp	*E. coli* fluorescence reporter for EsaR transcriptional activation	This study
ESN51 pesaR:GFP	*P. stewartii* fluorescence reporter for EsaR transcriptional repression	This study
ESN51 pPesaR-AC:gfp	*P. stewartii* fluorescence reporter for EsaR transcriptional activation	This study
JLD271 pJN105-expR2, prsmA:GFP	*E. coli* fluorescence reporter for ExpR transcriptional activation	This study

[a]Antibiotic resistances and working concentrations: 34 μg/mL chloramphenicol (Cm), 100 μg/mL ampicillin (Ap), 50 μg/mL kanamycin (Km), 10 μg/mL gentamycin (Gm), and 30 μg/mL nalidixic acid (Nal).

Table S2. Primers used in this study.

Primer	Sequence	Purpose
pJN105-sdiAec FWD for pJN105-esaR	CAGCGTCAGCAGCAAGGTAGTCTAGAGCGGCCGCCACCGC	construct pJN105-esaR
pJN105-sdiAec REV for pJN105-esaR	AGGAAGAAAGAGAACATCATGAATTCGCTAGCCCAAAAAAACG GGTATGG	
pET17b-esaR FWD for pJN105-esaR	TTTTTTGGGCTAGCGAATTCATGATGTTCTCTTTCTTCCTTGAAA A	
pET17b-esaR REV for pJN105-esaR	GCGGTGGCGGCCGCTCTAGACTACCTTGCTGCTGACGCTG	
pCS-pesaRlux FWD for pCS-pesaS-lux	CACTGGCGGCCGTTACTAGTCCGCAAAATGGATGGCAAAT	construct pesaSlux
pCS-pesaRlux REV for pCS-pesaS-lux	CACTGTTGTGAGCAAAAAGGGGTGAAGACGAAAGGGCCTC	
pPesaR-AC:gfp FWD for pCS-pesaS-lux	GAGGCCCTTTCGTCTTCACCCCTTTTTGCTCACAACAGTGT	
pPesaR-AC:gfp REV for pCS-pesaS-lux	ATTTGCCATCCATTTTGCGGACTAGTAACGGCCGCCAGTG	
pPesaR-AC:gfp FWD for pesaR:GFP	AAAGAAGCAGCGGATCCTCTGGATCCTCTAGATTTAAGAAGGA G	construct pesaR-GFP
pPesaR-AC:gfp REV for pesaR:GFP	CAATCTGCCTCGAGGTGAAGGTGAGCAAAAAGGATCCCCG	
pCS-PesaRlux FWD for pesaR:GFP	CGGGGATCCTTTTTGCTCACCTTCACCTCGAGGCAGATTG	
pCS-PesaRlux REV for pesaR:GFP	TTCTTAAATCTAGAGGATCCAGAGGATCCGCTGCTTCTTT	
pJN105-EsaR FWD for pJN-expR2	TCAAGCCTGAGCCTTTATAATCTAGAGCGGCCGCCACCG	construct pJN105-expR2
pJN105-EsaR REV for pJN-expR2	TCAGAGCAAAATACAGACATGAATTCGCTAGCCCAAAAAAACG GGTATGG	
Ecc71 FWD for pJN-expR2	TTTTTTGGGCTAGCGAATTCATGTCTGTATTTTGCTCTGAC	
Ecc71 REV for pJN-expR2	CGGTGGCGGCCGCTCTAGATTATAAAGGCTCAGGCTTGA	
pPesaR-AC:gfp FWD for prsmA-GFP	TACTTGTTAGTTTAGTCGATCGAAAGGGCCTCGTGATACG	construct prsmA:GFP
pPesaR-AC:gfp REV for prsmA-GFP	GGGGAGACAGAGAGACCCGCTCTAGATTTAAGAAGGAGATATA CATATGAG	
Ecc71 FWD for prsmA-GFP	TCTCCTTCTTAAATCTAGAGCGGGTCTCTCTGTCTCCCC	
Ecc71 REV for prsmA-GFP	CGTATCACGAGGCCCTTTCGATCGACTAAACTAACAAGTAGTG ACAAAC	

Table S3. Comparative antagonism assay data for AHLs in four receptors (LuxR, ExpR1, ExpR2, and EsaR).[a,b] Values >75% are highlighted in pink. Negative values indicate potential activators.

Compound	LuxR	ExpR1	ExpR2	EsaR-repressed (*pesaR*)	EsaR-activated (*pesaS*)
OHHL	0	0	0	0	0
BHL	43	48	19	-46	-22
HHL	69	67	12	-126	-96
OHL	86	78	38	-40	-95
DHL	96	91	76	-59	-11
dDHL	73	77	62	-27	-63
hDHL	27	4	7	-71	-11
OOHL	63	65	26	-138	-89
ODHL	47	42	33	-94	-69
OdDHL	86	85	73	-10	-45
OtDHL	5	4	7	-53*	-41*
**B7**	80	78	66	-36	-50
**B8**	34	37	23	-4	-4
**B9**	44	48	37	-10	-24
**B10**	48	57	49	17*	-6
**C1**	14	4	11	-61	-13
**C2**	59	64	23	-50	-14
**C3**	63	52	41	13	-24
**C4**	49	44	27	15*	-9
**C5**	75	78	48	2*	-3
**C6**	65	63	54	-58	-40
**C7**	53	57	41	-26	1
**C8**	68	73	59	-65	-43
**C9**	47	49	32	-14	-8
**C10**	85	73	68	25	-5
**C11**	78	83	63	-56	-57
**C12**	63	65	55	4*	-12
**C13**	47	44	32	12	-29
**C14**	-18	-15	-4	-3	-56
**C15**	23	27	3	0	-6
**E1**	78	74	62	2*	1*
**E2**	71	68	59	-42	0
**E7**	88	79	71	-3	-26
**E9**	0	4	16	-17	-67
**E12**	83	58	5	37	-10
**E13**	84	69	-6	-11	-2
**K3**	79	71	62	-76*	-24*

[a] Data for LuxR and ExpR1/2 are reproduced from reference 6. For LuxR, ExpR1, and ExpR2, compounds were tested at 5 μM against 5 μM OHHL. LuxR activation was measured by luminescence in *V. fischeri* ES114 (*ΔluxI*), and ExpR1 and ExpR2 activity was measured by cellulase activity in *P. versatile* (previously *P. carotovora*) AC5117 (*ΔexpI:ΔexpR2*) and *P. versatile* AC5099 (*ΔexpI:ΔexpR1*), respectively.[6] [b] EsaR activation was measured by luciferase activity or GFP production in an *E. coli* reporter; asterisks indicate that GFP reporter was used. Compounds were tested at 100 μM against the approx. EC_{50} for OHHL (330 nM for the EsaR-repressed luciferase reporter, 180 nM for the repressed GFP reporter and both activated reporters). For the activated reporter, percent antagonism was calculated as (signal/OD_{600}-EC_{50}[OHHL])/DMSO. For the repressed reporter, percent antagonism was calculated as 100-[(signal/OD_{600}-DMSO)/EC_{50}[OHHL]]. Values represent one biological replicate and at least 3 technical replicates.

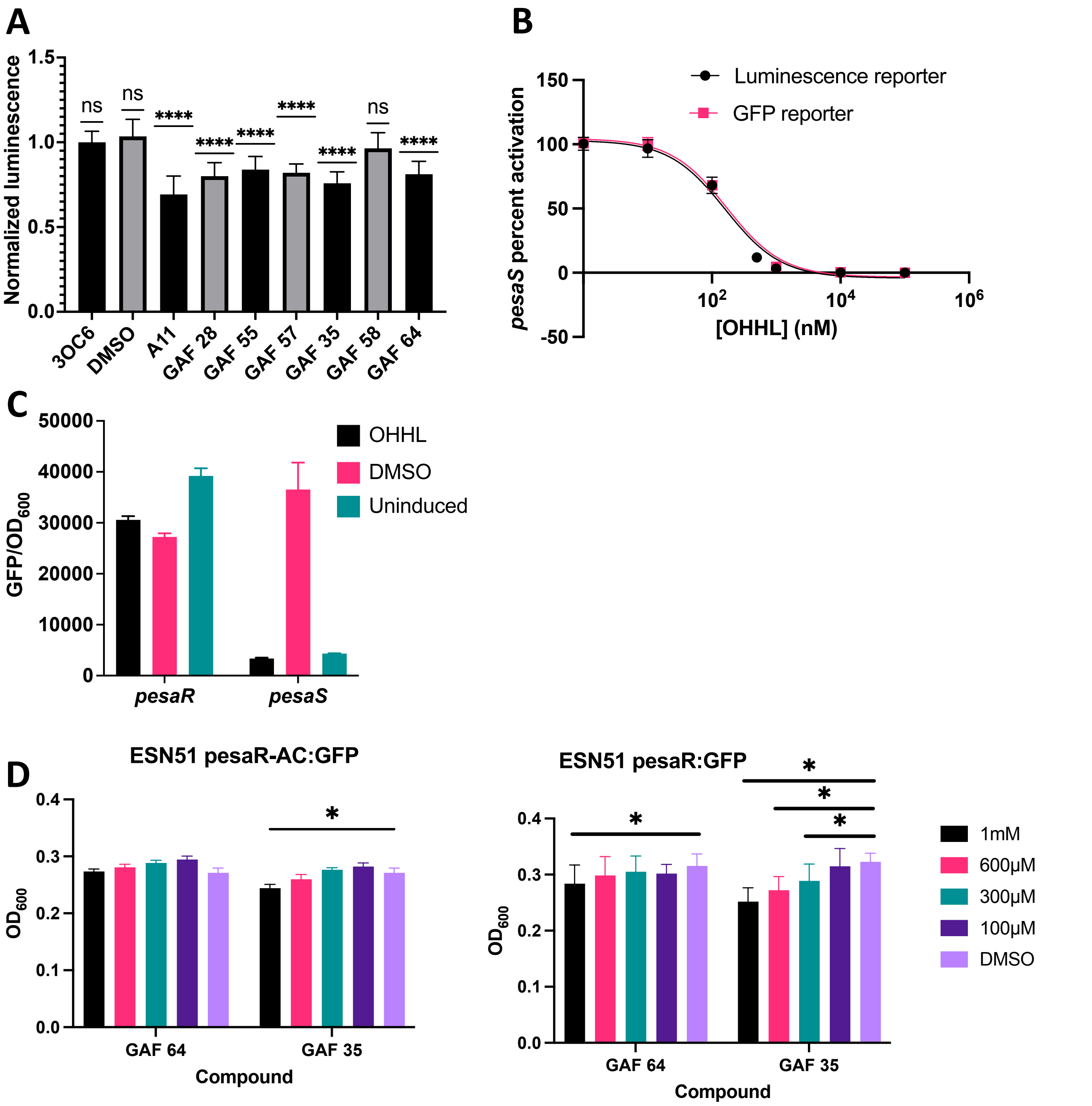

Figure S1. Luminescence inhibition, dynamic range, and growth of reporter strains. All data were collected as described for EsaR reporters in the main text Methods section. Error bars represent standard deviation. (A) Luminescence inhibition by selected compounds (3OC6 = OHHL). Compounds were tested at 1 mM in *E. coli* JLD 271 pCS-pesaRlux (no EsaR production plasmid). Data were normalized to JLD271 (no plasmids) with DMSO as 0% and JLD 271 pCS-pesaRlux with OHHL as 100%, and OD_{600} values were used to correct for small differences in growth. Normalized luminescence = (Lum/OD_{600}-JLD271)/OHHL. Results are an average of 3 biological replicates, each performed as 3 technical replicates. **** indicates p-values <0.0001. (B) *E. coli* EsaR-activated luciferase and GFP reporters produce similar results. Data were corrected for OD_{600} and normalized to 100 µM OHHL (0%) and DMSO (100%). Results are an average of 3 biological replicates, each performed as 3 technical replicates. (C) *E. coli* reporter with EsaR-repressed promoter *pesaR* has much lower dynamic range than reporter with EsaR-activated promoter *pesaS*. (D) Growth inhibition in *P. stewartii* ESN51 reporters for certain compounds (**GAF 64** and **GAF 35**). * indicates p-values <0.05.

A

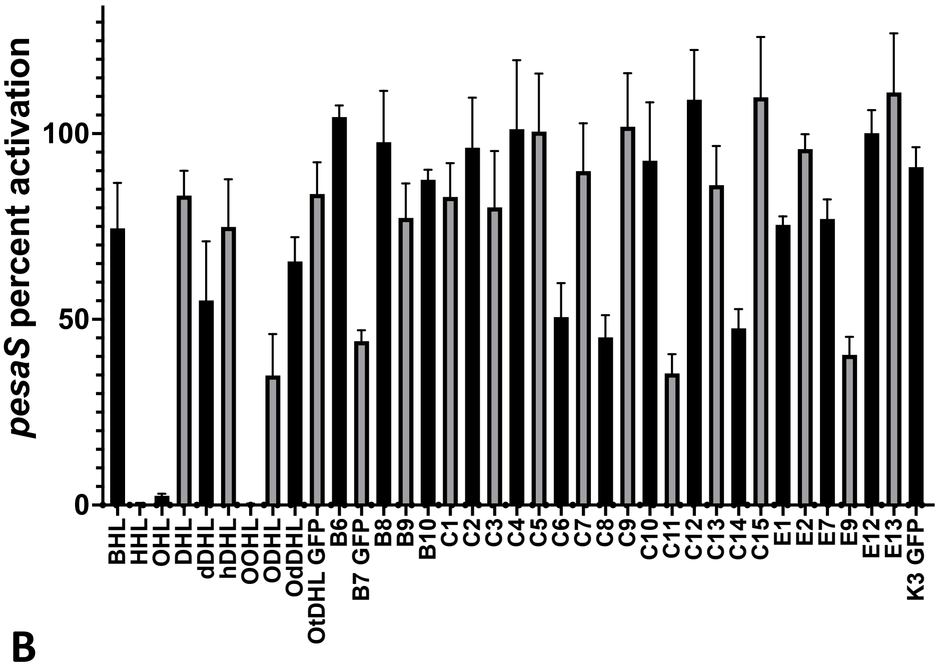
pesaS percent activation
100
50
0
BHL
HHL
OHL
DHL
dDHL
hDHL
OOHL
ODHL
OdDHL
OtDHL GFP
B6
B7 GFP
B8
B9
B10
C1
C2
C3
C4
C5
C6
C7
C8
C9
C10
C11
C12
C13
C14
C15
E1
E2
E7
E9
E12
E13
K3 GFP

B

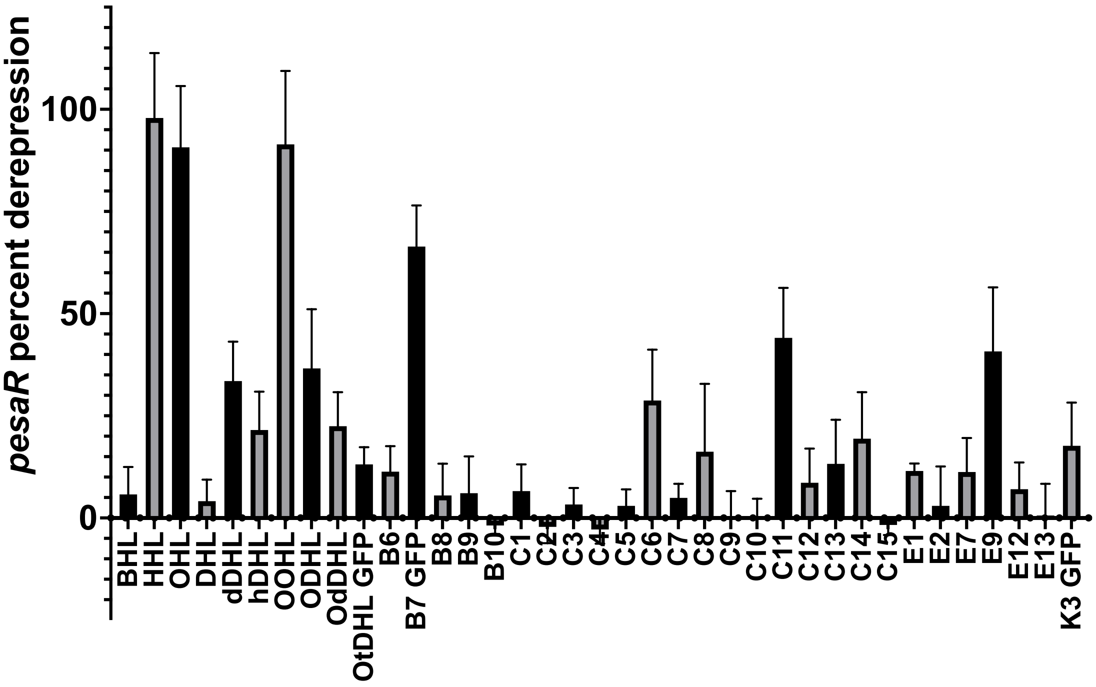
pesaR percent derepression
100
50
0
BHL
HHL
OHL
DHL
dDHL
hDHL
OOHL
ODHL
OdDHL
OtDHL GFP
B6
B7 GFP
B8
B9
B10
C1
C2
C3
C4
C5
C6
C7
C8
C9
C10
C11
C12
C13
C14
C15
E1
E2
E7
E9
E12
E13
K3 GFP

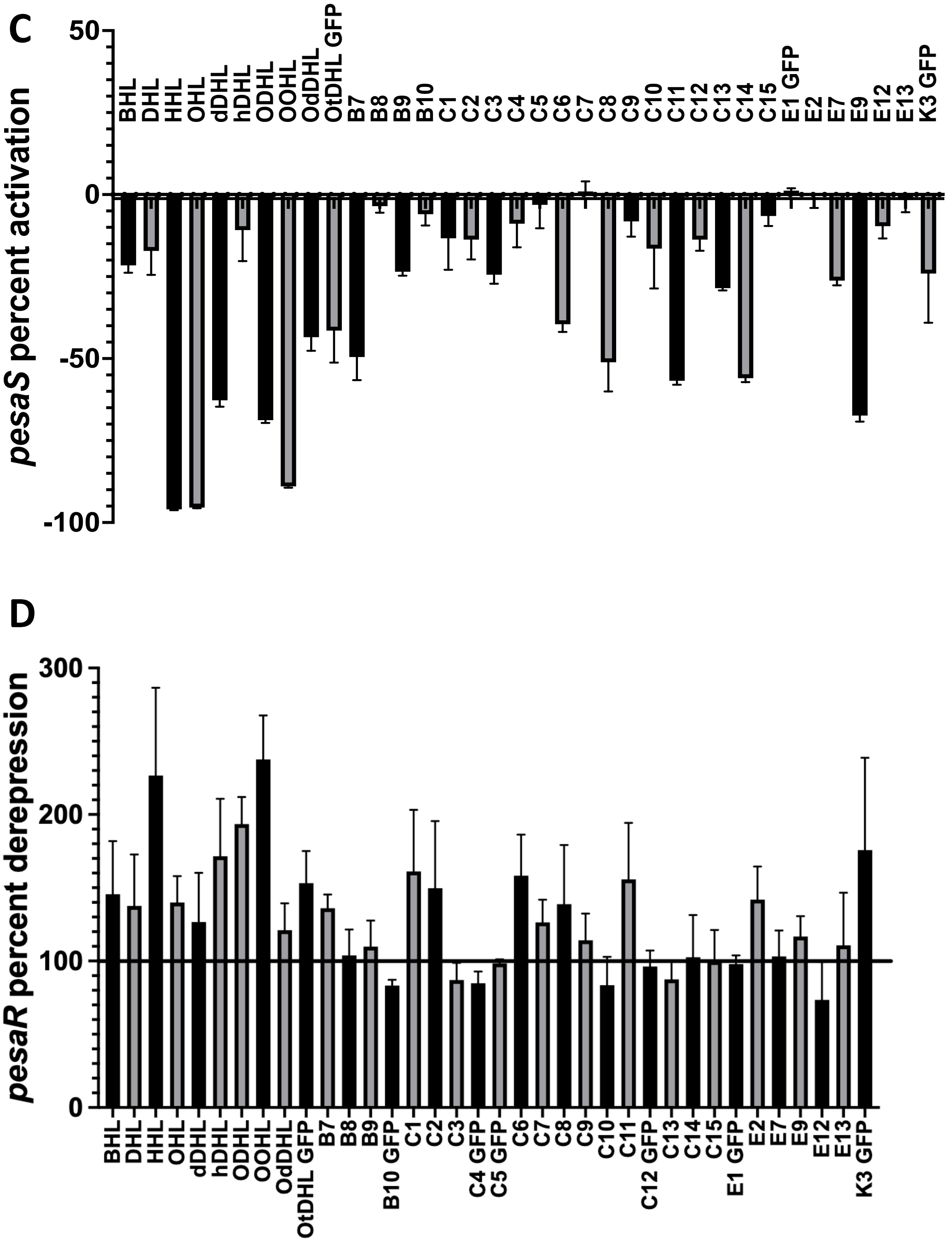

Figure S2. Agonism and antagonism primary screening data in EsaR for compounds previously tested in LuxR, ExpR1, and ExpR2 (data summarized in **Tables 1** and **S3**). Compounds were tested at 100 µM. Data obtained in the luciferase reporter unless "GFP" is indicated next to the compound name, and all data were normalized to OD_{600} to account for small differences in cell density. Error bars represent standard deviation. (A) Agonism for EsaR-activated promoter. Data were normalized to 100 µM OHHL (0%) and DMSO (100%). (B) Agonism for EsaR-repressed promoter. Data were normalized to 100 µM OHHL (100%) and DMSO (0%). (C) Antagonism for EsaR-activated promoter. Compounds were competed against 180 nM OHHL (approx. EC_{50}). Data were normalized to EC_{50} OHHL (0%) and DMSO (100%). Negative values indicate potential activators. (D) Antagonism for EsaR-repressed promoter. Compounds were competed against the approx. EC_{50} for each reporter (330 nM OHHL for the luciferase reporter or 180 nM for the GFP reporter). Data were normalized to EC_{50} OHHL (100%) and DMSO (0%). Values over 100% indicate activators.

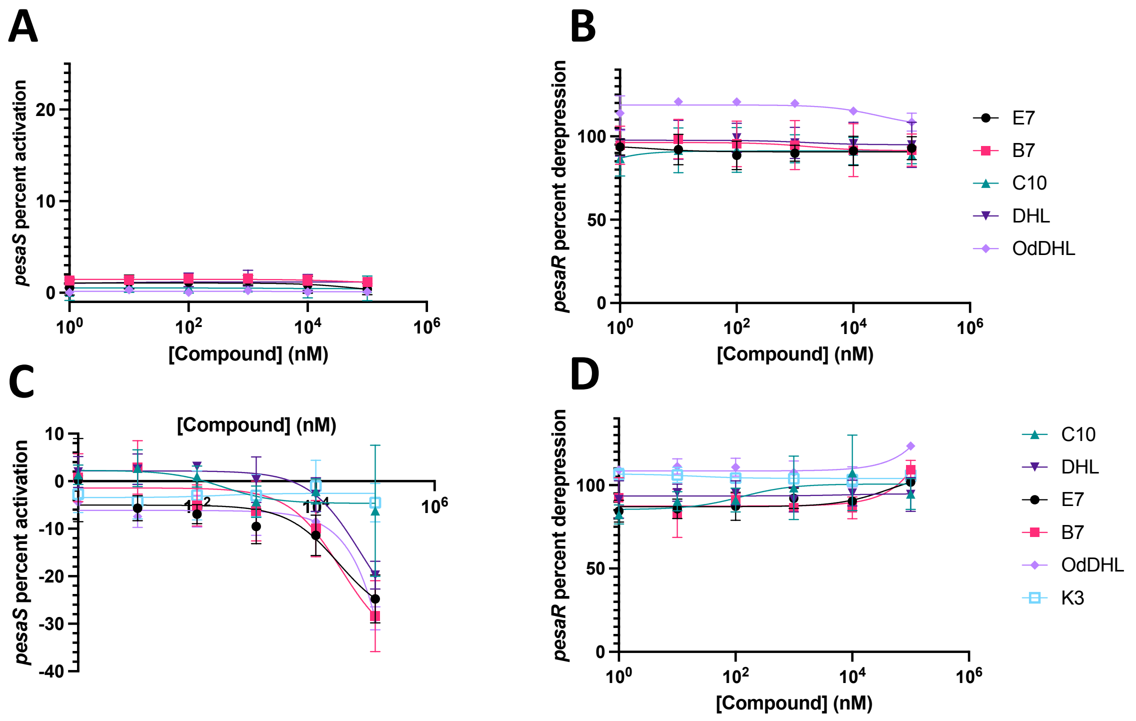

Figure S3. Antagonism dose-response data for selected compounds in *E. coli* GFP and *P. stewartii* EsaR reporters. All data were normalized to OD_{600}. Error bars represent standard deviation. (A) *E. coli* GFP reporter data with EsaR-activated promoter. Compounds were competed against 1650 nM OHHL (approx. EC_{90}). Data were normalized to EC_{90} OHHL (0%) and DMSO (100%). (B) *E. coli* GFP reporter data with EsaR-repressed promoter. Compounds were competed against 1650 nM OHHL (approx. EC_{90}). Data were normalized to EC_{90} OHHL (100%) and DMSO (0%). (C) *P. stewartii* reporter data with EsaR-activated promoter. Compounds competed against 115 nM OHHL (approx. EC_{50}). Data were normalized to EC_{50} OHHL (0%) and DMSO (100%). Negative values indicate activation. (D) *P. stewartii* reporter data with EsaR-repressed promoter. Compounds were competed against 155 nM OHHL (approx. EC_{50}). Data were normalized to EC_{50} OHHL (100%) and DMSO (0%).

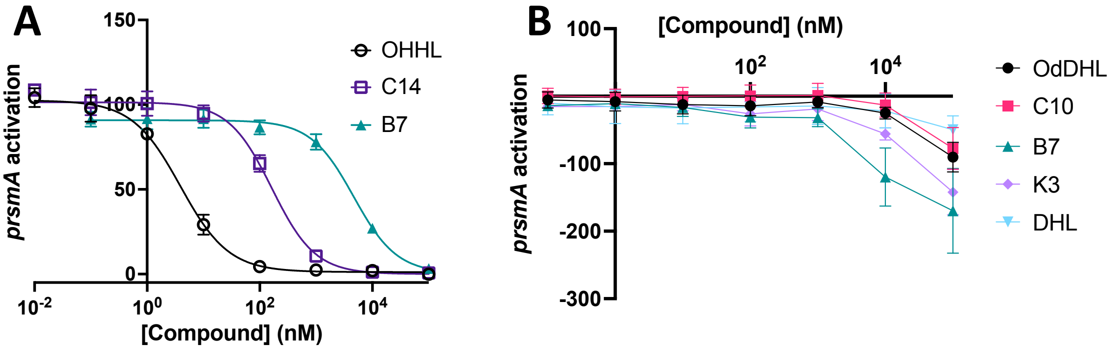

C

	**OHHL**	**C14**
EC_{50} *E. coli* (this study)	3.9 nM (95% CI 3.5-4.4 nM)	160 nM (95% CI 140-190 nM)
EC_{50} phenotypic reporter (ref. 6)	2 μM	0.5 μM

D

**Compound**	IC_{50} [μM]	Percent inhibition (5 μM)	Percent activation (200 μM)
OdDHL (**A4**)	0.75	73	17
DHL (**A9**)	0.90	76	44
**K3** (**B17**)	0.85	62	25
**C10** (**B18**)	0.90	68	19
**B7** (**C4**)	2.75	66	8

Figure S4. Screening data in *E. coli* reporter with ExpR2-activated promoter. All data were normalized to OD_{600} to account for small differences in cell density. Error bars represent standard deviation. (A) Agonism data, normalized to 100 μM OHHL (0%) and DMSO (100%). Data were corrected for OD_{600} and represent 3 biological and technical replicates for OHHL and **C14** or 2 biological replicates of 3 technical replicates for **B7**. (B) Antagonism data. Compounds were competed against 8 nM OHHL, and data were normalized to 8 nM OHHL (0%) and DMSO (100%). Data were corrected for OD_{600} and represent 2 biological replicates. Negative values indicate activation. (C) Comparison of EC_{50} values in *E. coli* reporter (dose-response curves shown in panel A) and previously reported phenotypic reporter data for OHHL and **C4** (from reference 6). (D) Summary of previously reported phenotypic reporter data for compounds shown in panel B (all data from reference 6). Compound numbering indicates naming in this study and that prior study (in paratheses).

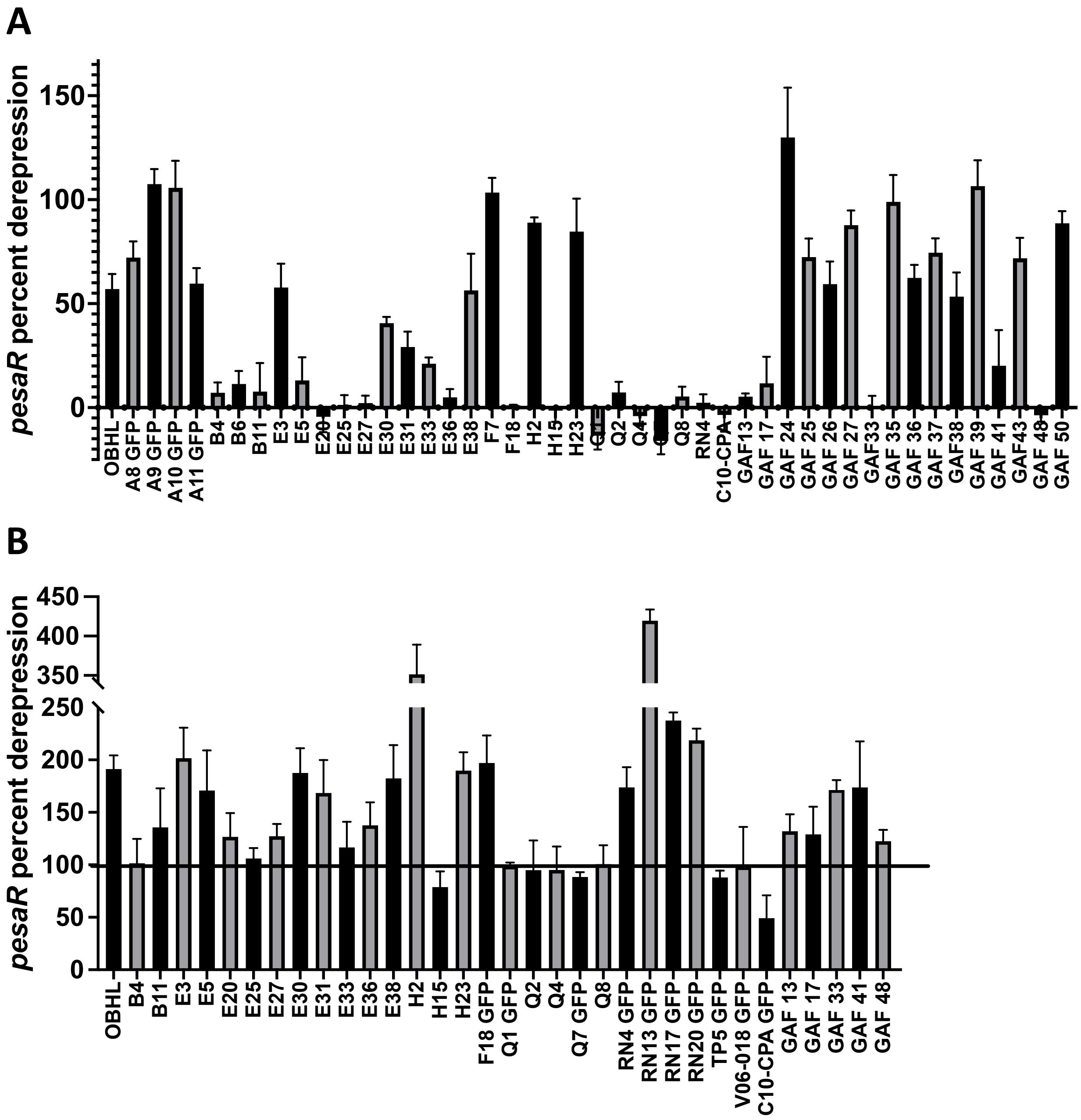

Figure S5. Representative agonism and antagonism primary screening data in the *E. coli* reporter with EsaR-repressed promoter. Data were obtained in the *E. coli* luciferase reporter unless "GFP" is indicated next to the compound name. All data were normalized to OD_{600}. Error bars represent standard deviation. See **Table S1** for strains and the main text Methods section for experimental details. (A) Agonism data. Data were normalized to 100 µM OHHL (100%) and DMSO (0%); percent agonism was calculated as (signal/OD_{600}-DMSO)/OHHL. (B) Antagonism data. Selected compounds were competed against OHHL at the approx. EC_{50} (330 nM for the luciferase reporter or 180 nM for the GFP reporter). Data were normalized to EC_{50} OHHL (100%) and DMSO (0%). Values over 100% indicate activators. The known LasR antagonists TP-5[7] and V-06-018[8] were included for comparison and found to be inactive as EsaR antagonists. C10-CPA, shown also to modulate LasR[9] and RhlR,[10] did not show antagonism in the EsaR-activated reporter and was not investigated further.

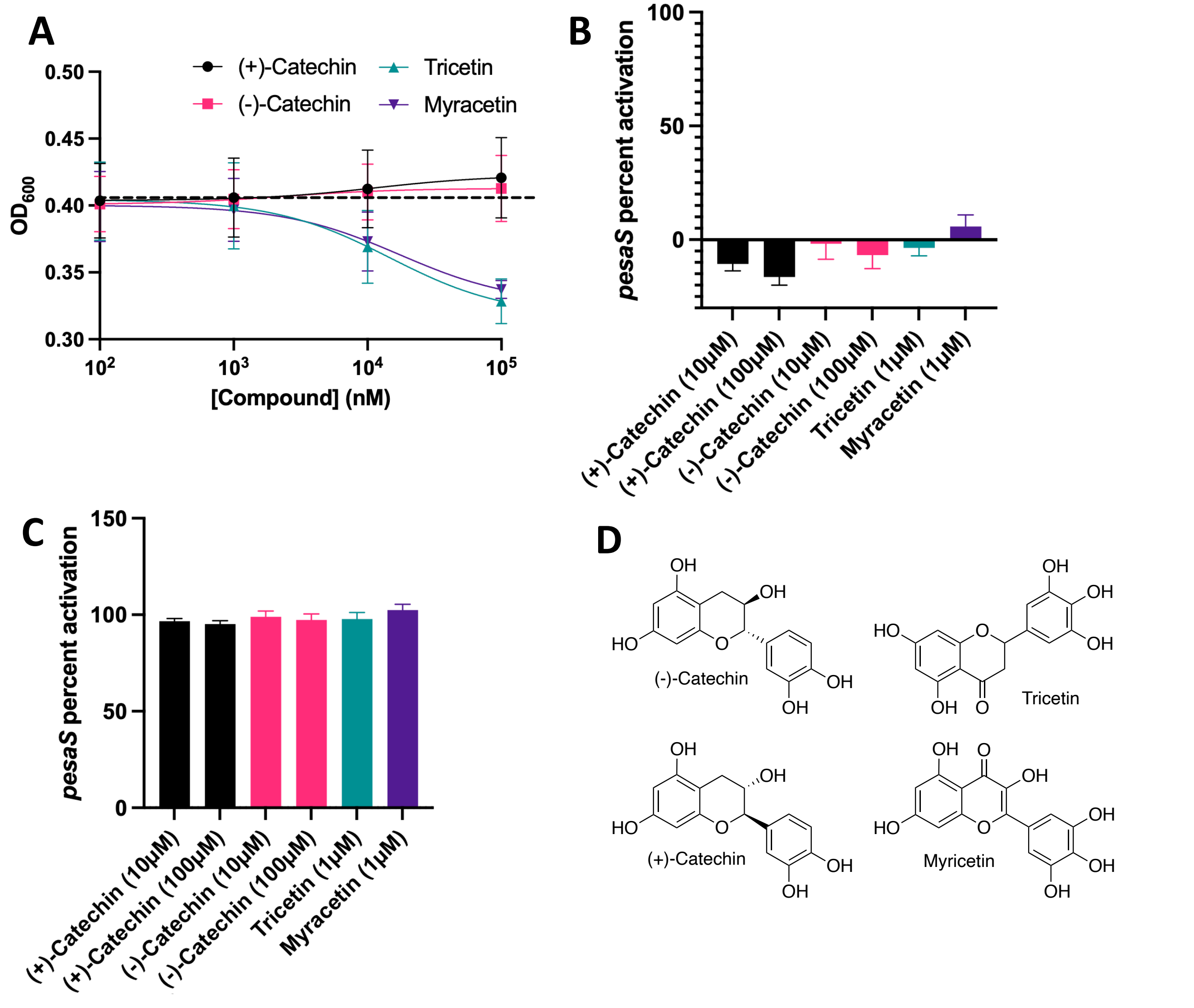

Figure S6. Evaluation of flavonoid compounds predicted by Neupane *et al.* to modulate EsaR[11] in the *P. stewartii pesaS* reporter. Error bars represent standard deviation. (A) Growth inhibition by flavonoid compounds. Dashed line indicates average OD_{600} of DMSO and OHHL control wells. Tricetin and myricetin were tested at 1 µM to avoid toxicity issues in reporter assays. (B) Antagonism data. Compounds were competed against 115 nM OHHL (approx. EC_{50}), and data were corrected for OD_{600}, normalized to 115 nM OHHL (0%) and DMSO (100%), and represent 2 biological replicates, each performed in technical triplicate. Negative values indicate potential activators. (C) Agonism data. Data were corrected for OD_{600}, normalized to 100 µM OHHL (0%) and DMSO (100%), and represent 2 biological replicates, each performed in technical triplicate. (D) Structures of tested compounds.

```
                              cov    pid   1 [         .         .         .         .         :         .         .         . 80
1 EsaR_P_stewartii        100.0% 100.0%     MFSFFLENQTITDTLQTYIQRKLSPLGSPDYAYTVVSKKNPSNVLIISSYPDEWIRLYRANNFQLTDPVILTAFKRTSPF
2 ExpR2_P_versatile_Ecc71  98.4%  52.8%     MSVFCSDNEIINNTIKSYLGRKLKQYGDLKYAYMIMNKKNPSQVVIISNYPQEWVNTYKENNYQHIDPVILTAINKVSPF

                              cov    pid  81         .         1         .         .         .         .         :         . 160
1 EsaR_P_stewartii        100.0% 100.0%     AWDENITLMSDLRFTKIFSLSKQYNIVNGFTYVLHDHMNNLALLSVIIKGNDQTALEQRLAAEQGTMQMLLIDFNEQMYR
2 ExpR2_P_versatile_Ecc71  98.4%  52.8%     SWEDNIVINSKLKFSKIFNLSKEYDIVNGYTFVLHDNNNNLAALSIMFEEHAPTDMENIVEENKDKLQMLLIAVHEKITT

                              cov    pid 161         .         .         .         2         .         .         .         . 240
1 EsaR_P_stewartii        100.0% 100.0%     LAGTEGERAPALNQSADKTIFSSRENEVLYWASMGKTYAEIAAITGISVSTVKFHIKNVVVKLGVSNARQAIRLGVELDL
2 ExpR2_P_versatile_Ecc71  98.4%  52.8%     LYK-EMTQSPQSRKQGDKEIFSQRENEILYWASMGKTYPEIALILDIKISTVKFHIGNVVKKLGVLNAKHAIRLGVELQL

                              cov    pid 241       ] 249
1 EsaR_P_stewartii        100.0% 100.0%     IRPAASAAR
2 ExpR2_P_versatile_Ecc71  98.4%  52.8%     IKPEPL---
```

Figure S7. Sequence alignment of EsaR and ExpR2 created using Clustal Omega with default parameters and visualized with Mview.[12] cov refers to sequence coverage and pid refers to percent sequence identity.

Dose-response agonism data in *E. coli* EsaR reporters.

Agonism dose-response curves in *E. coli* EsaR reporters corresponding to **Table 2** in the main text are shown below. All data were corrected for OD_{600} and normalized to 100 µM OHHL (0%) and DMSO (100%) for EsaR-activated reporters or 100 µM OHHL (100%) and DMSO (0%) for EsaR-repressed reporters. Data represents 3 biological replicates, each performed in technical triplicate, and error bars indicate standard deviation. See **Table S1** for strains and main text Methods for experimental details. Compound names are on the x-axis of each plot.

- **EsaR-activated GFP reporter**

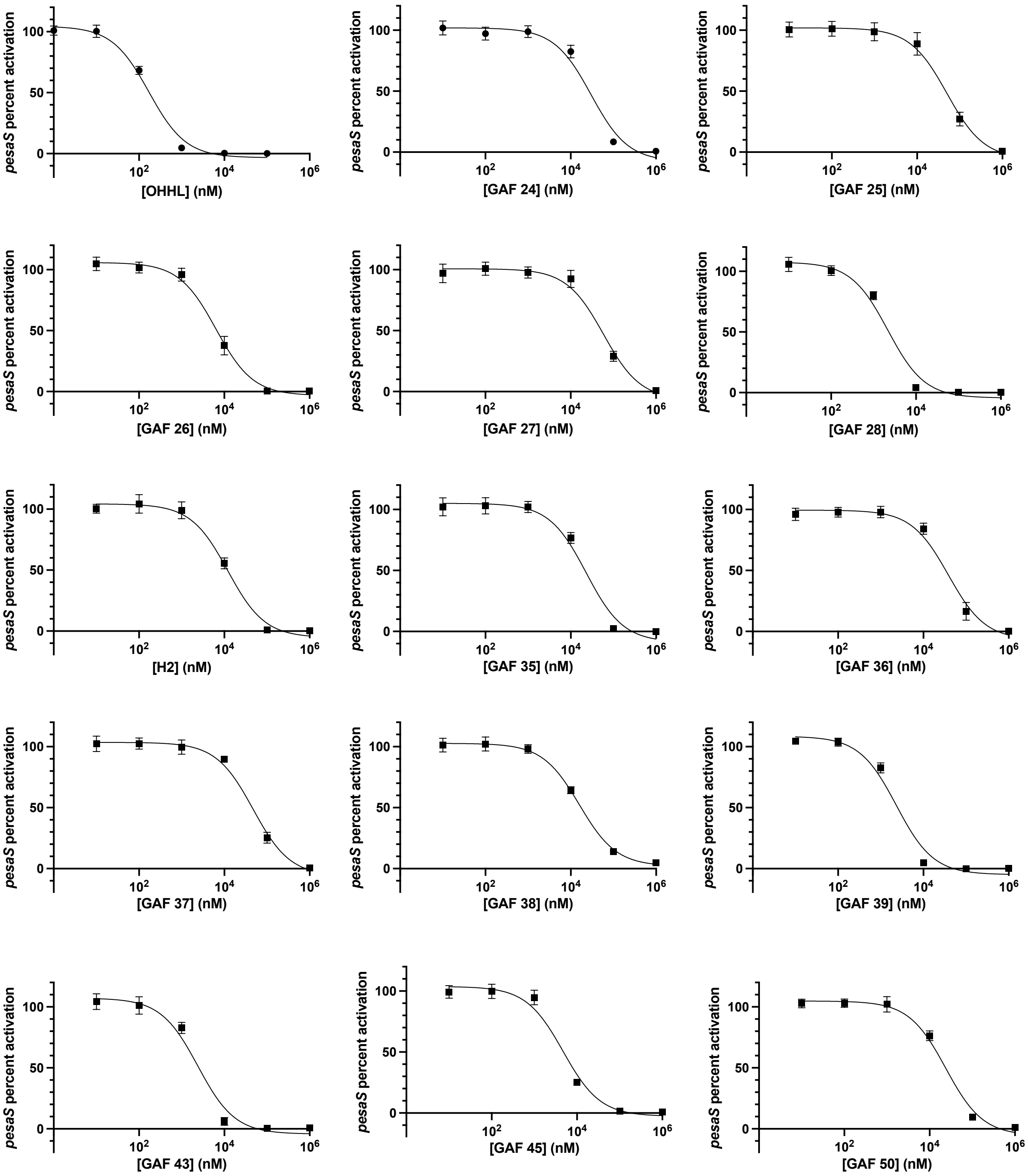

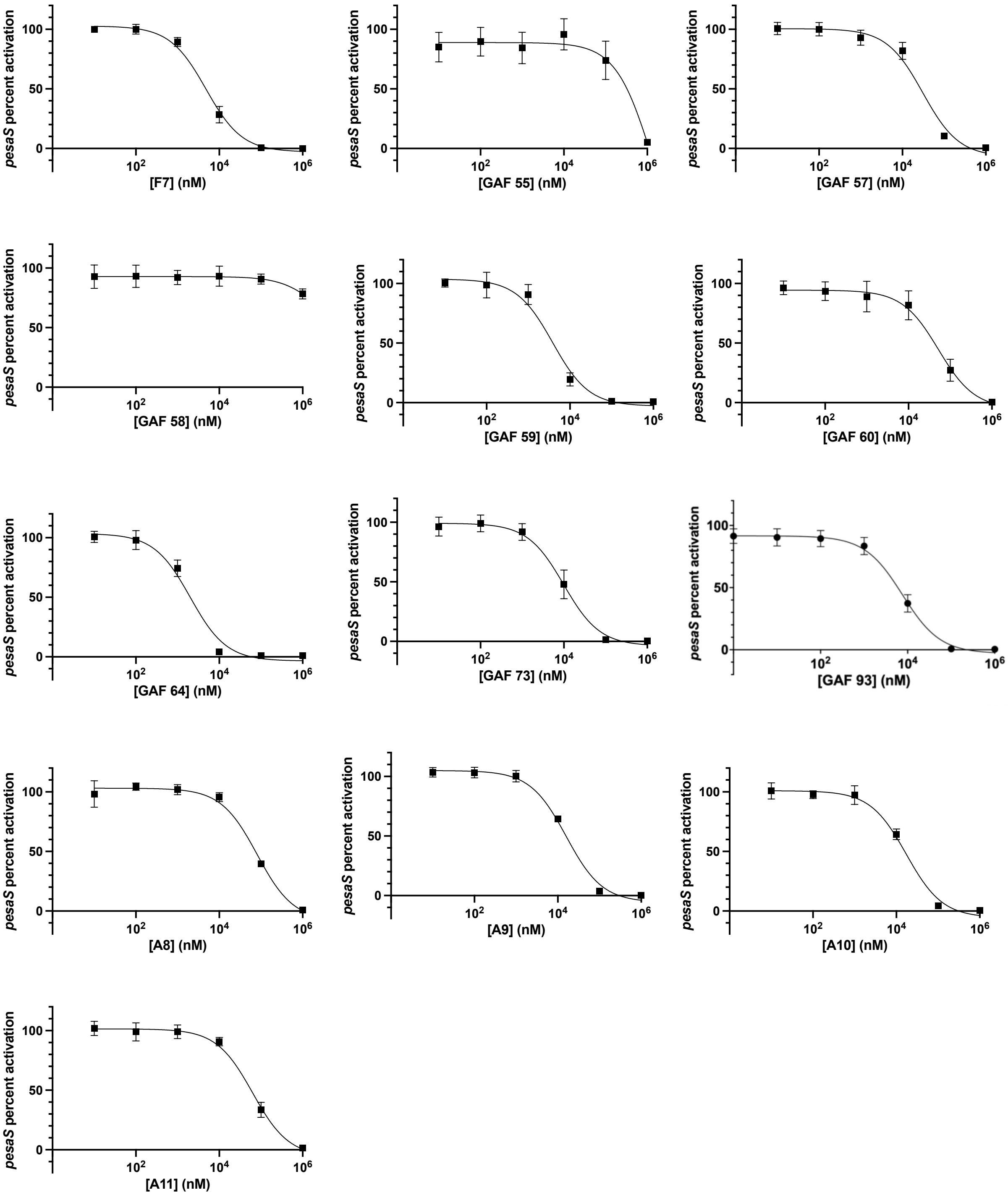

pesaS percent activation
100
50
0
10^2
10^4
10^6
[F7] (nM)
[GAF 55] (nM)
[GAF 57] (nM)
[GAF 58] (nM)
[GAF 59] (nM)
[GAF 60] (nM)
[GAF 64] (nM)
[GAF 73] (nM)
[GAF 93] (nM)
[A8] (nM)
[A9] (nM)
[A10] (nM)
[A11] (nM)

- **EsaR-activated luciferase reporter**

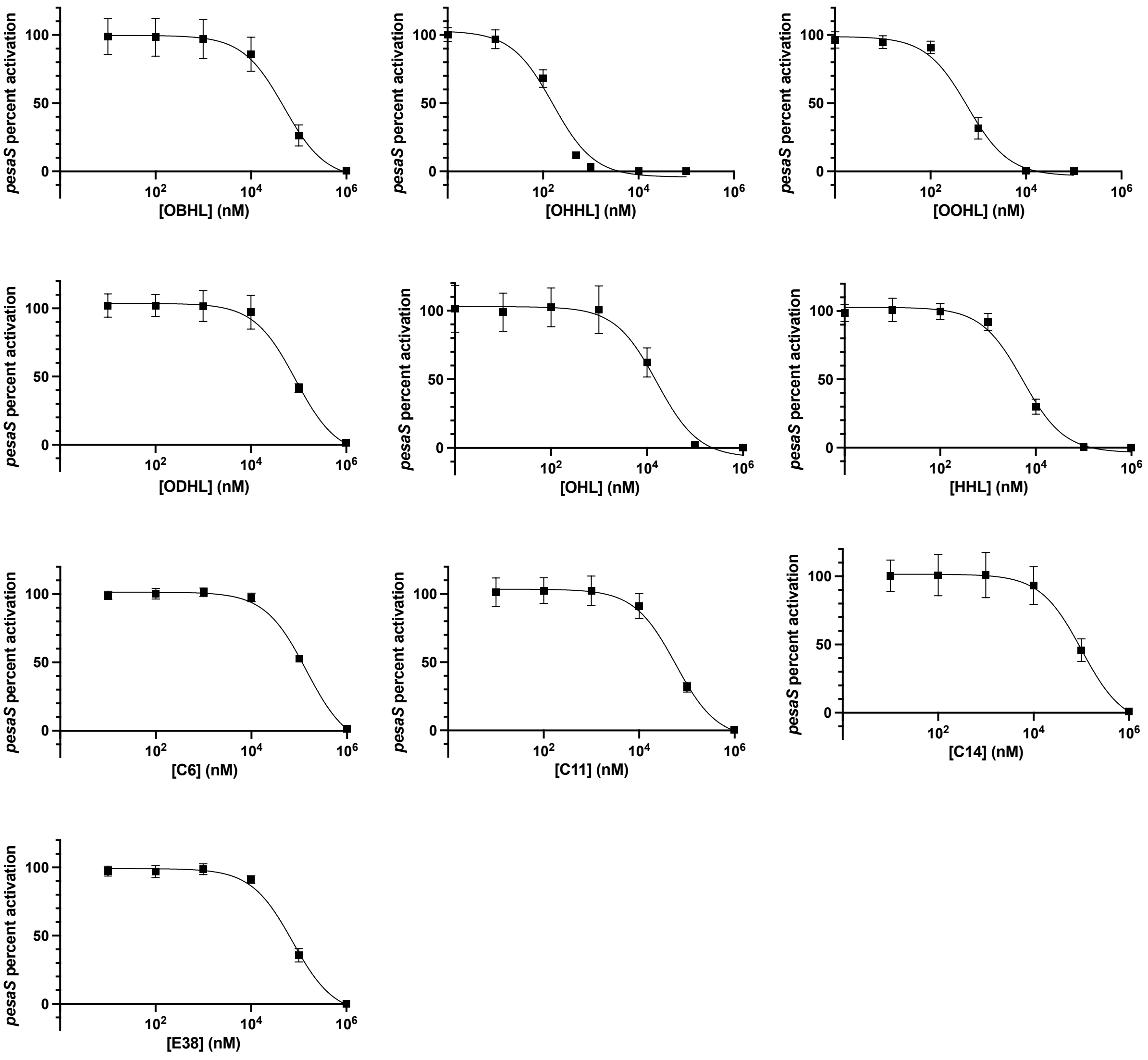

- **EsaR-repressed GFP reporter**

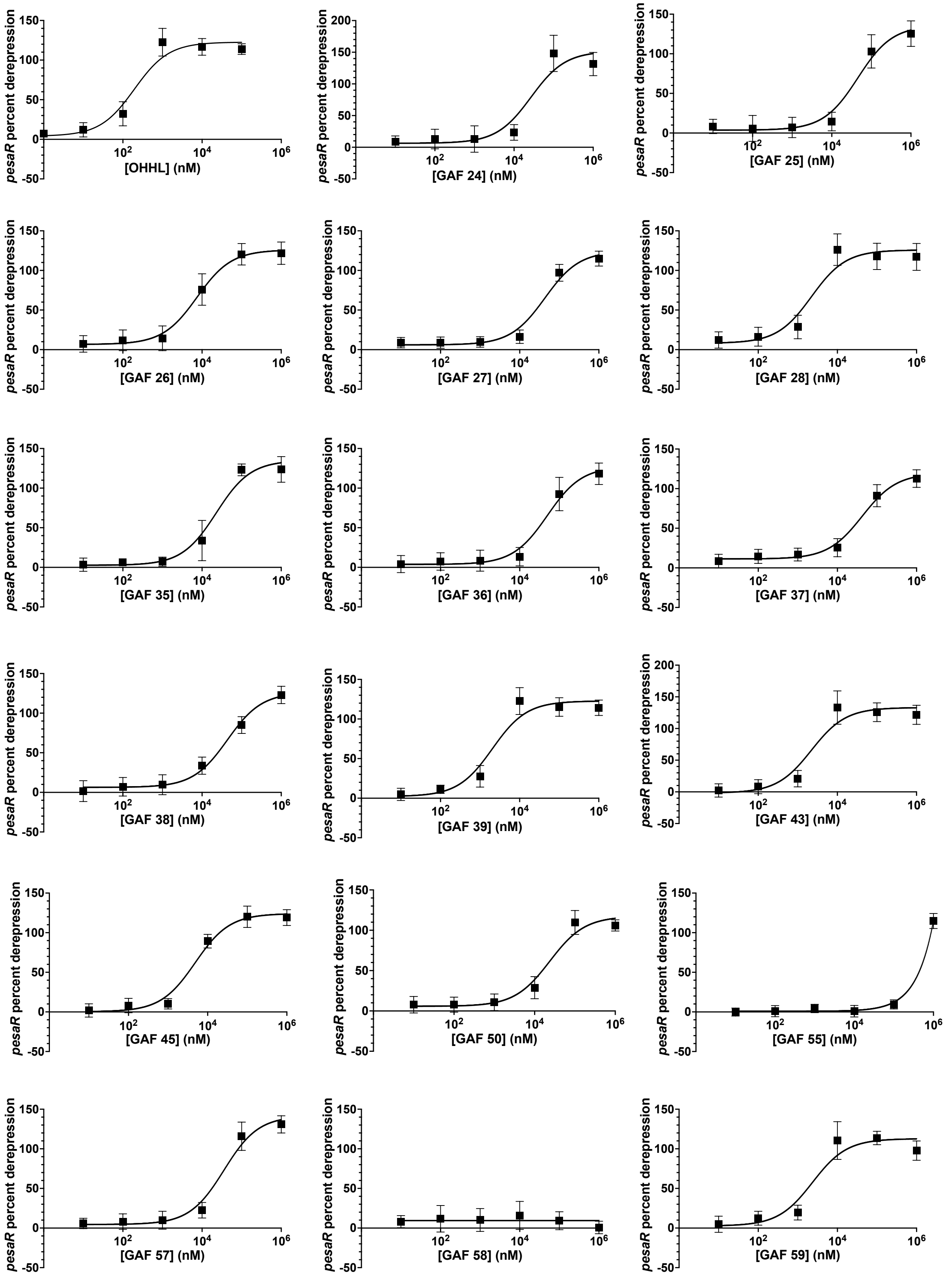

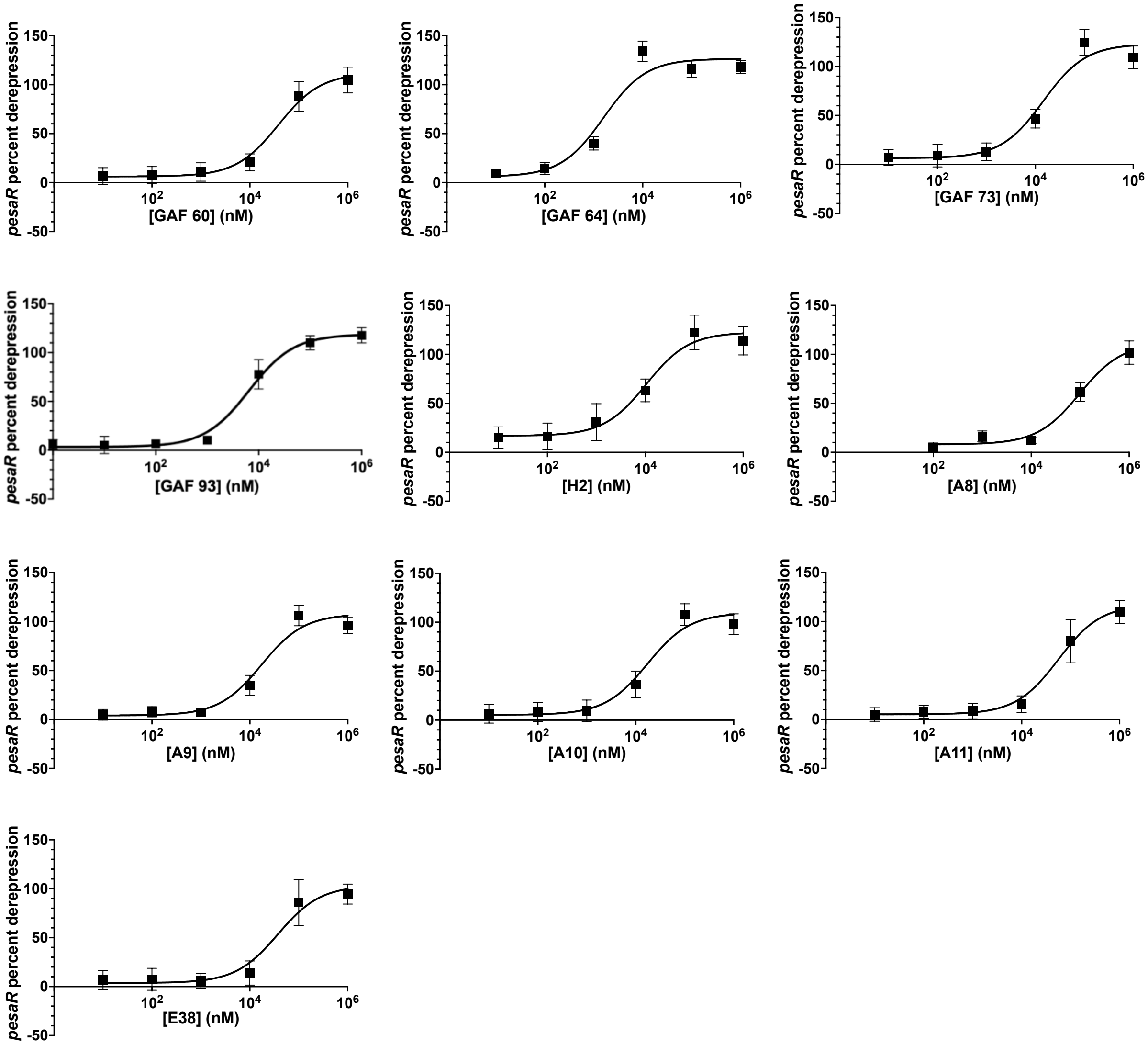
pesaR percent derepression
[GAF 60] (nM)
[GAF 64] (nM)
[GAF 73] (nM)
[GAF 93] (nM)
[H2] (nM)
[A8] (nM)
[A9] (nM)
[A10] (nM)
[A11] (nM)
[E38] (nM)

- **EsaR-repressed luciferase reporter**

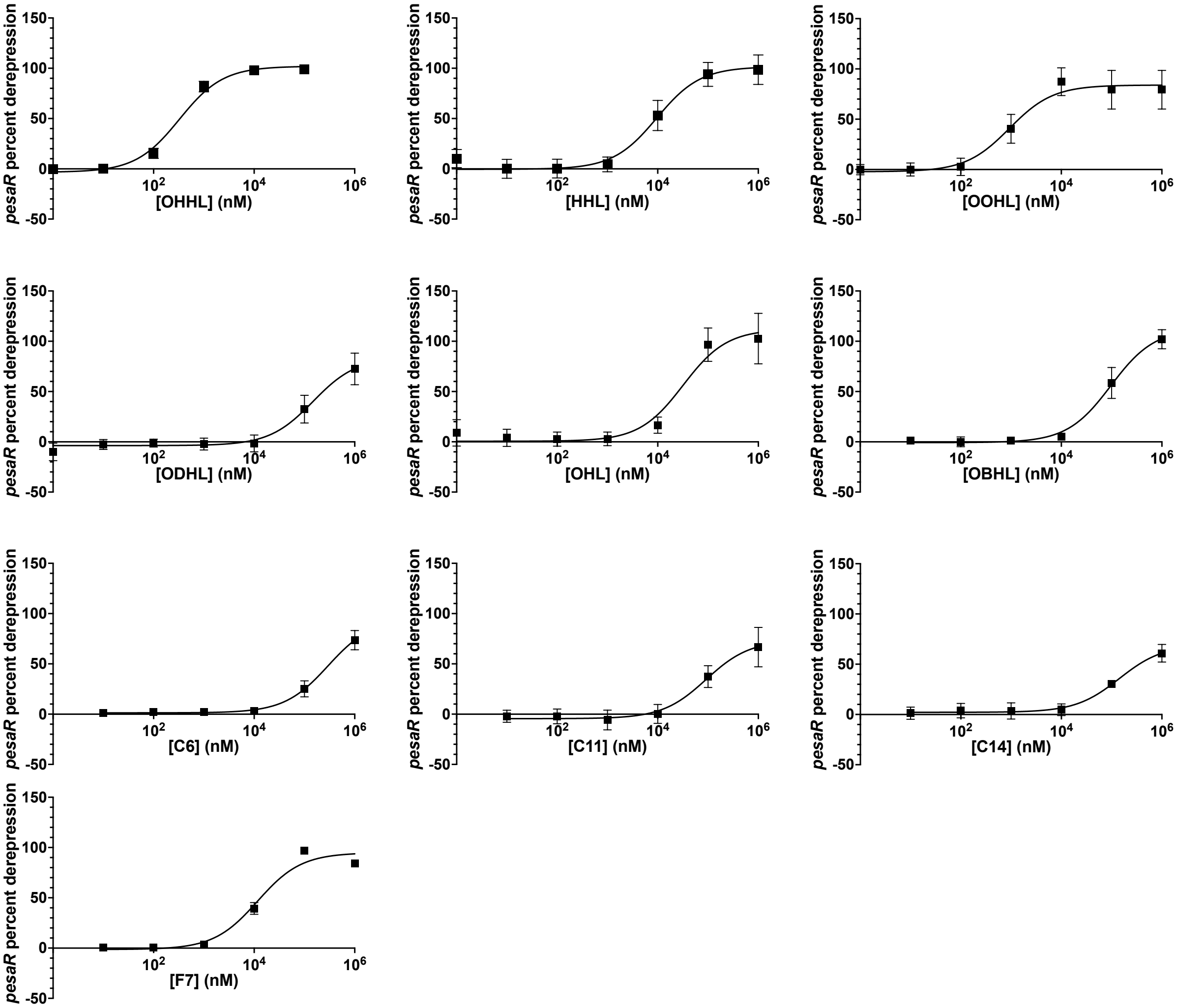

References.

[1] Cui, Y., Chatterjee, A., Hasegawa, H., and Chatterjee, A. K. (2006) *Erwinia carotovora* subspecies produce duplicate variants of ExpR, LuxR homologs that activate *rsmA* transcription but differ in their interactions with *N*-acylhomoserine lactone signals, *J. Bacteriol. 188*, 4715-4726. https://doi.org/10.1128/jb.00351-06

[2] Shong, J., Huang, Y. M., Bystroff, C., and Collins, C. H. (2013) Directed evolution of the quorum-sensing regulator EsaR for increased signal sensitivity, *ACS Chem. Biol. 8*, 789-795. https://doi.org/10.1021/cb3006402

[3] Schu, D. J., Carlier, A. L., Jamison, K. P., von Bodman, S. B., and Stevens, A. M. (2009) Structure/function analysis of the *Pantoea stewartii* quorum-sensing regulator EsaR as an activator of transcription, *J. Bacteriol. 191*, 7402-7409. https://doi.org/10.1128/jb.00994-09

[4] Lindsay, A., and Ahmer, B. M. (2005) Effect of sdiA on biosensors of *N*-acylhomoserine lactones, *J. Bacteriol. 187*, 5054-5058. https://doi.org/10.1128/jb.187.14.5054-5058.2005

[5] Simon, R., Priefer, U., and Pühler, A. (1983) A Broad Host Range Mobilization System for In Vivo Genetic Engineering: Transposon Mutagenesis in Gram Negative Bacteria, *Biotechnology (N. Y.) 1*, 784-791. https://doi.org/10.1038/nbt1183-784

[6] Palmer, A. G., Streng, E., Jewell, K. A., and Blackwell, H. E. (2011) Quorum sensing in bacterial species that use degenerate autoinducers can be tuned by using structurally identical non-native ligands, *ChemBioChem 12*, 138-147. https://doi.org/10.1002/cbic.201000551

[7] Müh, U., Hare, B. J., Duerkop, B. A., Schuster, M., Hanzelka, B. L., Heim, R., Olson, E. R., and Greenberg, E. P. (2006) A structurally unrelated mimic of a *Pseudomonas aeruginosa* acyl-homoserine lactone quorum-sensing signal, *Proc. Natl. Acad. Sci. U. S. A. 103*, 16948-16952. https://doi.org/10.1073/pnas.0608348103

[8] Müh, U., Schuster, M., Heim, R., Singh, A., Olson, E. R., and Greenberg, E. P. (2006) Novel *Pseudomonas aeruginosa* quorum-sensing inhibitors identified in an ultra-high-throughput screen, *Antimicrob. Agents Chemother. 50*, 3674-3679. https://doi.org/10.1128/AAC.00665-06

[9] Ishida, T., Ikeda, T., Takiguchi, N., Kuroda, A., Ohtake, H., and Kato, J. (2007) Inhibition of quorum sensing in *Pseudomonas aeruginosa* by *N*-acyl cyclopentylamides, *Appl. Environ. Microbiol. 73*, 3183-3188. https://doi.org/10.1128/aem.02233-06

[10] Nyffeler, K. E., Santa, E. E., and Blackwell, H. E. (2022) Small Molecules with Either Receptor-Selective or Pan-Receptor Activity in the Three LuxR-Type Receptors that Regulate Quorum Sensing in *Pseudomonas aeruginosa*, *ACS Chem. Biol. 17*, 2979-2985. https://doi.org/10.1021/acschembio.2c00675

[11] Neupane, S., Shah, H., Solanki, Z., Vyas, S., Modi, K., Parmar, P., and Goswami, D. (2020) A Walk into the EsaR Regulator of *Pantoea stewartii:* Inhibition of EPS Production By Interfering Quorum Sensing Pathway by Natural AHL Antagonist QS Inhibitors of EsaR, *Biosc. Biotech. Res. Comm. 13*, 150-158. https://doi.org/10.21786/bbrc/13.1specialissue/25

[12] Madeira, F., Madhusoodanan, N., Lee, J., Eusebi, A., Niewielska, A., Tivey, A. R. N., Lopez, R., and Butcher, S. (2024) The EMBL-EBI Job Dispatcher sequence analysis tools framework in 2024, *Nucleic Acids Res. 52*, W521-W525. https://doi.org/10.1093/nar/gkae241
